# Microfluidic chip systems for characterizing glucose-responsive insulin-secreting cells equipped with FailSafe kill-switch

**DOI:** 10.1186/s13287-024-04059-7

**Published:** 2024-12-18

**Authors:** Mohammad Izadifar, Mohammad Massumi, Kacey J. Prentice, Tatiana Oussenko, Biao Li, Judith Elbaz, Mira Puri, Michael B. Wheeler, Andras Nagy

**Affiliations:** 1 Lunenfeld-Tanenbaum Research Institute, Sinai Health System, Toronto, ON Canada; 2 Departments of Physiology, Temerty Faculty of Medicine, University of Toronto, Toronto, ON Canada; 3 Toronto General Hospital Research Institute, University Health Network, Toronto, ON Canada; 4 Australian Regenerative Medicine Institute, Monash University, Melbourne, VIC Australia; 5 Department of Obstetrics & Gynecology, Faculty of Medicine, University of Toronto, Toronto, ON Canada

**Keywords:** Stem cells, Beta cells, Type 1 diabetes, Cell therapy, Cell reprogramming, Organ on chip, Microfluidic systems, GSIS assay, Suicide gene

## Abstract

**Background:**

Pluripotent cell-derived islet replacement therapy offers promise for treating Type 1 diabetes (T1D), but concerns about uncontrolled cell proliferation and tumorigenicity present significant safety challenges. To address the safety concern, this study aims to establish a proof-of-concept for a glucose-responsive, insulin-secreting cell line integrated with a built-in FailSafe kill-switch.

**Method:**

We generated β cell-induced progenitor-like cells (βiPLCs) from primary mouse pancreatic β cells through interrupted reprogramming. Then, we transcriptionally linked our FailSafe (FS) kill-switch, HSV-thymidine kinase (TK), to Cdk1 gene using a CRISPR/Cas9 knock-in strategy, resulting in a FailSafe βiPLC line, designated as FSβiPLCs. Subsequently we evaluated and confirmed the functionality of the drug-inducible kill-switch in FSβiPLCs at different ganciclovir (GCV) concentrations using our PDMS-based transcapillary microfluidic system. Finally, we assessed the functionality of FSβiPLCs by characterizing the dynamics of insulin secretion in response to changes in glucose concentration using our microfluidic perfusion glucose-stimulated insulin secretion (GSIS) assay-on- chip.

**Results:**

The βiPLCs exhibited *Ins1*, *Pdx1* and *Nkx6.1* expression, and glucose responsive insulin secretion, the essential properties of pancreatic beta cells. The βiPLCs were amenable to genome editing which allowed for the insertion of the kill-switch into the 3’UTR of Cdk1, confirmed by PCR genotyping. Our transcapillary microfluidic system confirmed the functionality of the drug-inducible kill-switch in FSβiPLCs, showing an effective cell ablation of dividing cells from a heterogeneous cell population at different ganciclovir (GCV) concentrations. The Ki67 expression assessment further confirmed that slow- or non-dividing cells in the FSβiPLC population were resistant to GCV. Our perfusion glucose-stimulated insulin secretion (GSIS) assay-on-chip revealed that the resistant non-dividing FSβiPLCs exhibited higher levels of insulin secretion and glucose responsiveness compared to their proliferating counterparts.

**Conclusions:**

This study establishes a proof-of-concept for the integration of a FailSafe kill-switch system into a glucose-responsive, insulin-secreting cell line to address the safety concerns in stem cell-derived cell replacement treatment for T1D. The microfluidic systems provided valuable insights into the functionality and safety of these engineered cells, demonstrating the potential of the kill-switch to reduce the risk of tumorigenicity in pluripotent cell-derived insulin-secreting cells.

**Supplementary Information:**

The online version contains supplementary material available at 10.1186/s13287-024-04059-7.

## Introduction

Diabetes mellitus is a debilitating disease characterized by elevated blood glucose levels due to insufficient insulin production by pancreatic beta cells or insulin resistance in specific organs and tissues responsible for removing excess glucose from the blood. Type 1 diabetes (T1D) is an autoimmune form of the disease that causes the destruction of pancreatic beta cells. The current treatments involve manual of pump delivered exogenous insulin injections to manage glucose levels. However, the glucose regulation provided by these methods is far from as accurate as what beta cells can achieve, resulting in frequent hypo- and hyperglycemic events. Consequently, patients can experience severe complications such as retinopathy, neuropathy, nephropathy, and cardiovascular disease [1, 2].

T1D provides an ideal target for cell replacement therapies. The clinical feasibility of this approach has already been demonstrated through the successful development of the “Edmonton Protocol” for islet transplantation [3, 4]. This protocol involves the infusion of cadaveric islets into the portal vein of the patient’s liver, followed by immune suppression to prevent rejection of the transplanted allogeneic tissue. However, limitations such as the scarcity of sufficiently matching cadaveric islet donors, the challenges of long-term graft survival, and the need for immune suppression make this procedure far from ideal [5].

The in vitro generation of functional insulin-secreting cells from human pluripotent cells (hPSCs) holds great promise in providing an abundant supply of donor β-like cells. As such, there has been significant progress in developing methods to guide the differentiation of hPSCs to pancreatic β-like cells [6, 7]. However, early protocols report relatively low efficiency in producing the islet-comprising cell types, mainly glucose-responsive insulin-secreting β cells, typically ranging from around 10% to 40% [8–10]. This has resulted in the need to purify [6, 11, 12] therapeutic cells or further optimize differentiation techniques. In addition, as a rule, therapies that use cells grown and differentiated in vitro have raised safety concerns due to the unintended formation of tumorigenic cells [13] in the competitive space during extensive in vitro cell growth. An additional diabetes-related safety concern is the potential for unregulated insulin secretion by the transplanted cells, which can cause hypoglycemia, resulting in loss of consciousness, seizures and life-threatening consequences [14, 15].

A third major limitation is the necessity for systemic immunosuppression to prevent the patient’s immune system from targeting and destroying the transplanted cells. This immune suppression weakens the patient's ability to generate an effective immune response, thereby heightening their vulnerability to infections and a range of cancers. As a result, while the transplanted cells are safeguarded, the patient's overall health and safety are significantly compromised [16–18].

To overcome some of the limitations, we opted to utilize the several-week-long reprogramming process, which is capable of reverting somatic cells back into the pluripotent embryonic stage [19]. A deductive logic led us to define two transition points in the reprogramming process: the point-of-no-return (PNR) and the commitment point to induced pluripotent stem (iPS) cells (CPS) [20]. PNR marks the stage where cells lose their original identity and, beyond that, cannot go back – as a default—to the originating cell type if the reprogramming is stopped. By stopping reprogramming beyond PNR but before CPS, we were able to generate induced multipotent lung progenitor-like cells (iPLCs) from alveolar type II cells of the lung [21]. Beyond CPS, on the other hand, the cells are committed to the pluripotent state and continue proceeding to become fully reprogrammed iPS cells without the need for reprogramming factors.

Several efforts have also been made to address the safety issues [22–25]. We have developed our own safety system [26], designated as FailSafe kill-switch, which permits the extremely reliable elimination of potentially tumorigenic, uncontrolled-manner-dividing cells from a transplant. It also allowed the quantitation of potential risks associated with cell therapies in the function of a number of cells that need to be transplanted into a patient. In addition, this system can also be used to select for non-dividing therapeutic cells either before or after grafting [26, 27], which could further increase the safety of cell therapy. Furthermore, recent progress in genome editing techniques has made it possible to induce hypoimmunogenicity [28–31], eliminating the need for systemic immune suppression, which further increases the safety of therapies [32].

Here, we report the design and use of microfluidic systems to initially characterize a cell line generated through interrupted reprogramming of primary pancreatic β cells of a mouse. These cells retained the ability to secrete insulin in a glucose-responsive manner and allowed clonal genome editing to introduce our FailSafe kill-switch system.

## Materials and methods

### Generation of pancreatic β cell-specific doxycycline (dox)-inducible reprogrammable mouse model

**Fig. 1:**
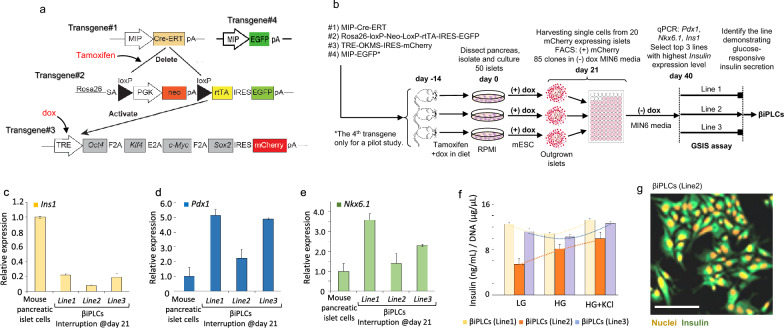
Schematics of generation and initial characterization of βiPLCs. **a** Quadruplet transgenic mice enable On/Off expression of the reprograming Yamanaka factors only in β cells of the pancreas. (See text for more details.) **b** The experimental design to generate βiPLCs. The relative expression of hallmarks **c** insulin, **d** *Pdx1* and **e** *Nkx6.1* of βiPLCs compared to the mouse pancreatic islet cells (control), measured by quantitative RT-PCR, **f** GSIS assay reveals that only line 2 exhibits regulated insulin secretion in response to low and high glucose concentration, **g** immunostaining of insulin expressed by βiPLCs line 2 (scale bar: 20 μm)

We generated the dox-inducible β-cell-specific reprogrammable mice through the crossbreeding of the following three transgenic mouse lines, resulting in triple transgenic animals: (1) a mouse insulin promoter (MIP)-driven tamoxifen-inducible Cre recombinase mouse [33], JAX: Stock No. 024709; producing Cre recombinase only in insulin expressing cells. (2) our ROSA26-loxP-neo-loxP-rtTA-IRES-GFP [34], JAX: Stock No. 005670; Upon tamoxifen-induced activation of Cre recombinase, these mice express rtTA from the Rosa26 locus. (3) PB:TetO-OKMS-mCherry [35], JAX: Stock No. 031009, where the OKMS cassette, encodes the four Yamanaka factors (Oct4, Klf4, c-Myc, and Sox2) linked to an mCherry reporter [35] (Fig. 1a).

Following tamoxifen activation on the rtTA expression from the Rosa26 locus, the administration of dox initiates and sustains reprogramming by inducing the expression of the randomly integrated polycistronic OKMS. A fourth transgene and MIP-EGFP line [36] (JAX: Stock No. 006864) was used only in an informative pilot experiment described in the Results section. The work has been reported in line with the ARRIVE guidelines 2.0.

### Pancreatic islet isolation and culture during reprogramming of β-cells

Three four-month-old triple-transgenic mice were fed with a tamoxifen and dox diet two weeks prior to harvesting their pancreas. We euthanized the mice by using carbon dioxide (CO_2_) in a euthanasia chamber for 10 min at a flow rate of 3 L/min, allowing for a CO_2_ fill rate of 30% of cage volume per minute, which meets the Canadian Council on Animal Care (CCAC) welfare standards. Anesthesia was not used in this study. The death of the euthanized mice was confirmed by checking for heartbeat and respiration before proceeding to harvest their pancreas tissues for isolating their islets using a published protocol [37]. The protocol was reviewed and approved by the ethics board of The Centre for Phenogenomics (TCP) (AUP 0182). Fifty islets from each mouse were dispersed and cultured in a 10-cm dish per mouse in the mouse primary islet culture media for one day (RPMI media supplemented with FBS (15%), penicillin–streptomycin (1%), MEM non-essential amino acid (1%), sodium pyruvate (1%), beta-mercaptoethanol (55 μM), and GlutaMAX (1%)) [38] containing dox (1 μg/ml). Then, we replaced the media with mES media (DMEM media supplemented with FBS (15%), LIF (1000 U/mL), penicillin–streptomycin (1%), MEM non-essential amino acid (1%), sodium pyruvate (1%), beta-mercaptoethanol(55 μM), and GlutaMAX (1%)) containing dox (1 μg/ml). The culture medium was refreshed every 2 days. Dox was removed from the media when the reprogramming expression was no longer wanted. Confocal fluorescence video microscopy (Zeiss, LMS 750) was used to follow the expression of the fluorescent reporters (mCherry for OKMS expression) in addition to EGFP for the islets derived from the four-transgenic mouse [see Additional files 1, 2, 3].

### Generation of insulin-secreting βiPLCs using the interrupted reprograming

On day 21, we harvested cells grown from 20 mCherry expressing islets on each dish, dissociated them with trypsin–EDTA treatment that was optimized for mouse ES cells, and then FACS sorted to isolate mCherry-positive cells from each islet outgrowth separately. We seeded mCherry positive cells from each islet onto separate wells of the 96-well plate and obtained 85 lines cultured in mouse insulinoma (MIN6) culture media [39] for three weeks without dox. Then, we measured the expression of *Ins* using RT-qPCR in each line and selected the top three with the highest insulin expression level [see Additional file 1a]. Then, we checked the expression of *Pdx1* and *Nkx6.1* using RT-qPCR and compared them to those of the primary islets. Also, we measured glucose-stimulated insulin secretion (GSIS) and then identified the line demonstrating the best glucose-responsive insulin secretion, which was then designated as the βiPLC line for the rest of the study.

### Generating FailSafe βiPLCs (FSβiPLCs)

We transcriptionally linked Cdk1 and Herpes simplex virus thymidine kinase (HSV-TK) –mCherry in βiPLCs using CRISPR/Cas9 assisted knock-in strategy, following our earlier publication describing the FailSafe system [26]. The genetically edited βiPLCs were isolated by treating cells with puromycin as the puromycin resistance gene in the construct allowed for positive selection. The selected puromycin-resistant βiPLCs were transfected with the Cre recombinase-containing plasmid to remove the stop codon (puromycin resistance gene). It resulted in linking Cdk1 and HSV-TK-mCherry to establish the safety bio-switch in βiPLCs, designated as FSβiPLCs. The mCherry expressing FSβiPLCs were single-cell plated in a 96-well plate by FACS (MoFlo Astrios EQs Sorter, Beckman Coulter, IN, USA) and cultured. A replica plate was treated with GCV (1 µM) for seven days to test for efficient killing of FSβiPLCs. PCR genotyping of all clones to test the expected knock-in into Cdk1 led to identifying clone#81, showing that the suicide gene (TK) was inserted into the 3’UTR of Cdk1 in a hemizygous manner.

### GCV sensitivity of FSβiPLCs in 2D culture

FSβiPLCs were plated in 2D culture at a density of 2 × 10^4^ cells/well in a 96-well culture plate and incubated for 24 h. Subsequently, the cells were treated with GCV in the culture medium at five different concentrations (0, 1, 20, 50 μM) for 10 days, with the culture medium being refreshed daily. The cell viability corresponding to each concentration of GCV was assessed at days 0, 2, 4, 7, and 10. A FailSafe mouse embryonic stem cell (FSmESC) [26] served as a positive control for GCV-mediated ablation of dividing cells.

### Microfluidic-based assessment of GCV sensitivity in a 3D microenvironment

A transcapillary-resembling microfluidic system [see Additional file 4] was developed using soft lithography technique [see Additional files 5 and 6] to test GCV sensitivity of FSβiPLCs in a 3D microenvironment. The transcapillary diffusion of GCV was validated experimentally [see Additional files 5 and 7]. Cells were harvested and mixed with ultra pure low viscosity guluronate (UPLVHG) sodium alginate (NOVAMATRIX, Sandvika, Norway) (1.8% (w/v) at 3 × 10^6^ cells/mL, and then injected (20 μL/min) into the central chamber of the microdevice followed by incubation for 10 min at 37 °C. Next, the microfluidic system was connected to a culture medium circuit integrated with a confocal microscope (LSM750, Zeiss) equipped with an incubation chamber [see Additional file 4] before pumping culture medium (20 μL/min) into the microchannels for 48 h. Then, DAPI (Sigma-Aldrich, Cat#D9564) was added to the circulating medium (1:15,000), to stain necrotic cell while leaving live cells unstained [40]. Next, fluorescent time-lapse imaging was conducted for βiPLCs and FSβiPLCs at GCV concentrations of 0, 1, 20, and 50 μM. This range of GCV concentrations is relevant to peak plasma levels in mice, reported between 3.9 and 47.1 μM in pharmacokinetic studies [41]. Time-lapse images were analyzed in ImageJ, and a temporal average apparent cell viability index was calculated [see Additional file 5]. After 7 days, cells were retrieved by injecting alginate lysate (500 μg/mL) into the side microchannels to break down alginate and wash out the cells from the microchamber. Cells were separated using a 40 μm mesh strainer, washed with culture medium, and used for the cell viability assay via flow cytometry.

### Insulin secretion measurement using a perfusion GSIS assay on-a-chip

A PDMS-based perfusion GSIS assay on-a-chip was designed, fabricated, computationally modeled and validated [see Additional files 5 and 8] to assess insulin secretion from βiPLCs and FSβliPLCs in real-time. The device was coated with Geltrex (ThermoFisher Scientific) followed by cell seeding within the microchannels. Then, microchannels were washed with Krebs–Ringer Bicarbonate (KRB) buffer for 30 min at 37 °C before connecting the device inlets to three syringes containing glucose solutions at 2 mM (LG), 20 mM (HG) and KCl/HG KRB. Sequentially, starting with LG, followed by HG, and finally KCl (30 mM)/HG in KRB, the solutions were injected into the device at 10 μL/min at 37 °C. Samples (100 μL) were collected at the sampling port every 10 min for ~ 45 min before switching the inlet. A waiting time of ~ 12 min, as predicted by our Computational Fluid Dynamics (CFD) model [see Additional file 8], was implemented for each sampling interval whenever switching inlets from LG to HG. Following each sampling, the samples were promptly centrifuged at 450 RCF for 5 min, and 80 μL of the supernatant was transferred into a V-bottom 96-well plate placed on ice for insulin content measurement using the Insulin Ultra Sensitive assay kit (Revvity HTRF® technology, Canada) for each time point in triplicate.

### Cell viability assessment

Cells were washed once in PBS and then resuspended in Annexin V binding buffer (cat# 422201, BioLegend) at 1 million cells/mL. Then cells were stained by adding 5 μL fluorochrome-conjugated Annexin V-APC (Cat# 640920, BioLegend) to 100 μL of the cell suspension for 15 min at room temperature. After washing the cells with 2 mL of binding buffer, they were resuspended in 200 µL of binding buffer including dead cell marker DAPI (Sigma-Aldrich, Cat#D9564). Flow cytometry was performed using a Beckman Coulter Gallios flow cytometer. The signals corresponding to the cells stained with DAPI and Annexin V-APC were detected in channels FL9 and FL6, respectively. Dead cells from ethanol treatment were used as the negative control. Data was analyzed with Kaluza software (Beckman Coulter).

### Proliferation assay

Ki67 proliferation assay was performed according to the manufacturer’s instruction for the flow cytometry. Briefly, the cells were trypsinized, collected, centrifuged (1000 rpm for 5 min) and then washed twice in the FACS buffer (1% BSA in PBS) at days 0, 4, and 7. Then, cells were permeabilized with ice-cold 70% ethanol (1 mL per million cells) followed by incubation at 4 °C for 1 h. Following two washes with the FACS buffer, the cells were stained with 5 μL Alexa Fluor 488-conjugated anti-mouse Ki67 mAb (BioLegend, Cat#151,204) or isotype control (BioLegend, Cat#400,625) for 30 min at room temperature in the dark. After 2 washes with FACS buffer, cells were resuspended in the FACS buffer containing DAPI (Sigma-Aldrich, Cat#D9564). Flow cytometry was performed using BD LSRFortessa X-20 cell analyzer (BD Biosciences) and data were analyzed using FlowJo software.

### Genomic and quantitative PCR

Total RNA and DNA were extracted using GenElute™ Mammalian Total RNA Miniprep Kit and DNase kit (Sigma-Aldrich) for qPCR and genomic PCR, respectively. Additional file 9 provides a list of primers utilized for evaluating gene expression levels via qPCR. The insertion of the TK gene was verified using genomic PCR, examining both the inside and outside of the Cdk1-TK at the 5’ and 3’ junctions. Insulin and HSV-TK mRNA levels were assessed using one-step real-time RT-qPCR with RNA-direct™ SYBR-Green Real-time PCR Master mix (Toyobo, Osaka, Japan) following the manufacturer’s protocol. Amplification was carried out on an ABI Prism 7500 Real-time PCR apparatus (Applied Biosystems, USA). GAPDH was used as the housekeeping gene for normalization in all RT-qPCR analyses.

### Immunostaining of insulin and Ki67

Cells were first washed with PBS and then fixed with a 4% paraformaldehyde (PFA) solution. Subsequently, cell permeabilization was carried out by treating the cells with a 0.1% Triton X-100 solution (Sigma-Aldrich) for 20 min at room temperature, followed by washing with PBS. The cells were then treated with a 5% w/v solution of Bovine Serum Albumin (BSA) for 2 h at room temperature. Next, cells were incubated with the primary anti-insulin antibody (ab7842, abcam) at a concentration of 5 μg/mL in a 0.025% Triton X-100 solution for 3 h at room temperature. After washing with PBS, cells were incubated with the secondary antibody (1:200) (ab150185) for 1 h at room temperature in the dark. For Ki67 staining, following permeabilization, cells were incubated with Alexa Fluor 488-conjugated anti-mouse Ki67 monoclonal antibody (BioLegend, Cat#151204) at a concentration of 0.25 μg per million cells in 100 μL of 0.025% Triton X-100 solution for 2 h at room temperature. Subsequently, cells were washed with PBS and imaged using confocal microscopy (LSM750, Zeiss, Germany).

### Statistical analysis

The paired Student's t-test and one-way analysis of variance were applied for comparison between two or more groups, respectively. We used SPSS software (IBM SPSS Statistics, Ver. 21.0. Armonk, NY: IBM Corp.) to perform the statistical analyses.

## Results

### Generating multi-transgenic mice allowing beta cell-specific reprogramming

To allow beta cell-specific reprogramming, we intercrossed four transgenic mouse lines. These transgenes and their interactions are illustrated in Fig. 1a. Transgene #1 contains a mouse insulin promoter (MIP) driven Cre-ERT recombinase [33], expressed exclusively in insulin-positive cells and excises the targets at the loxP sites on Transgene#2 in a tamoxifen-inducible manner (Fig. 1a). Transgene #2 expresses rtTA-IRES-EGFP from the ubiquitous Rosa26 locus [34] in a Cre recombinase excision-conditional manner. Transgene #3 is obtained from the tetracycline-inducible-reprogrammed Rep1 mice [35], which is a highly efficient reprogramming line. This dox-inducible polycistronic transgene encodes for all Yamanaka reprogramming factors (Oct4, Klf4, Nanog and c-Myc), reprogramming factors and is linked to an mCherry reporter. This triple transgenic animal was the source of cell line in the main body of experiments and data reported in the study. The transgene #4 (Fig. 1a) was introduced, and then the quadruple transgenic animals were only used in a pilot experiment to determine the time point when the expression of reprogramming factors had to be stopped to prevent the loss of critical features of their original state that needed to be retained. Transgene #4 is a MIP promoter-driven EGFP [36], reporting insulin expression with strong EGFP expression [see Additional file 1]. It's important to note that Transgene #2 also expresses EGFP after Cre excision. The expression is very weak, and this trace amount does not interfere with the readout provided by Transgene #4.

### Generation of permanent cell lines from β cells by interrupted reprogramming

Quadruplet transgenic mice were treated, as described in the Methods, prior to dissecting their islets on day 0 (Fig. 1b). From day 1, the isolated islets were cultured in ES cell media supplemented with dox to express the reprogramming factors [see Additional file 1]. During culture, the EGFP signal was monitored indicating the mouse insulin promoter activity, which became severely reduced but still detectable after three weeks in dox [see Additional files 1, 2, 3]. This information led us to decide to interrupt the reprogramming process on day 21 in the next experiment using three triple transgenic animals as donors (Fig. 1b)., following the experimental design described in the Methods. After testing the lines obtained on day 40 for insulin expression we selected the three highest expressors and quantitated their *Pdx1*, and *Nkx6.1* by qRT-PCR. Then, we tested their glucose responsiveness of insulin secretion. Interestingly, the lowest insulin-secreting line (line 2) demonstrated glucose responsiveness and responded to direct cellular depolarization with the secretagogue KCl (Fig. 1f). Immunohistochemistry also confirmed the expression of insulin in this line (Fig. 1g). Therefore, line #2 was selected for further studies and designated as the βiPLC line, which stands for beta cell induced progenitor-like cell line.

It is worth noting that the total insulin secretion (Fig. 1f) across LG, HG, and HG + KCl conditions correlates with the insulin expression levels (Fig. 1c) for all three lines; however, only line 2 exhibits GSIS response. Notably, despite higher *Pdx1* and *Nkx6.1* expression (Fig. 1d, e), lines 1 and 3 lacked GSIS response, whereas line 2, with lower *Pdx1* and *Nkx6.1* expression, exhibited glucose responsiveness. This might be explained by changes in the expression levels of these transcription factors during beta cell differentiation. Studies have shown that *Pdx1* and *Nkx6.1* are highly expressed in progenitor cells but decreased as the cells mature into fully functional beta cells [42–44]. Therefore, the higher expression level of *Pdx1* and *Nkx6.1* in lines 1 and 3 could indicate earlier progenitor stage [45], which may explain their lack of GSIS response.

### Generating FailSafe βiPLC (FSβiPLC) line

**Fig. 2:**
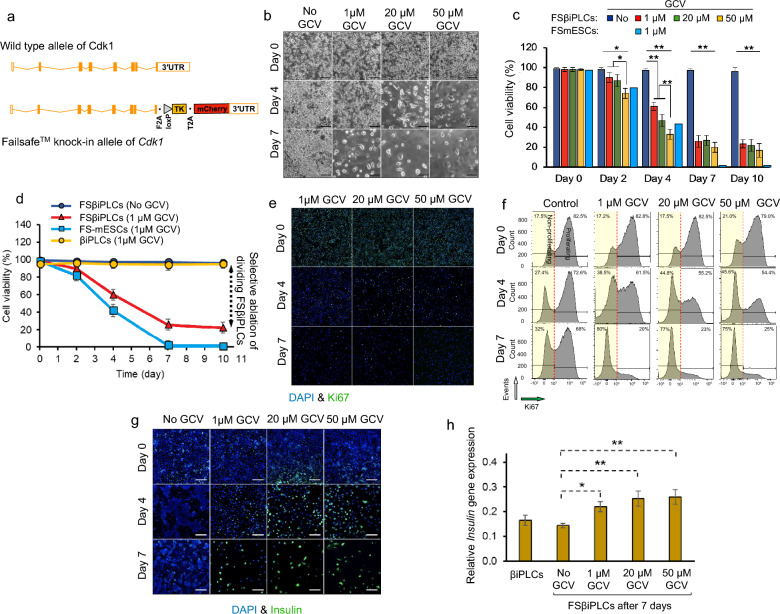
**a** the structure of Failsafe™ knock-in allele of Cdk1 consisting of the suicide gene TK and mCherry reporter compared to the wild type allele of Cdk1. **b** the representative phase contrast images of FSβiPLCs visualize changes in cell density over 6 days under GCV treatment at 0 (control), 1, 20 and 50 μM GCV (scale bar: 100 μm), **c** results from the cell viability assay of FSβiPLCs for GCV concentrations of 1, 20 and 50 μM over 10 days compared to the non-treated FSβiPLCs (control), where **d** GCV treatment (1 μM) leads to the complete ablation of FSmESCs (dividing cells, control), in contrast a certain population of FSβiPLCs survives the GCV treatment which represents the selective ablation of FSβiPLCs, **e** FSβiPLCs stained with anti-Ki67 antibody (green) and DAPI (blue) to visualize the distribution of Ki67 expressing cells at days 0, 4 and 7 during the GCV treatment of FSβiPLCs at 1, 20 and 50 µM GCV concentrations. **f** The quantitative evaluation of proliferating and non-proliferating FSβiPLCs represented by flow cytometry analysis of Ki67 protein presentation by FSβiPLCs treated by 1, 20 and 50 µM GCV compared to non-treated FSβiPLCs (control) at days 0, 4 and 7. **g** immunohistochemistry images show insulin expression by FSβiPLCs corresponding to GCV treatment at 1, 20 and 50 μM in days 0, 4 and 7 compared to non-treated FSβiPLCs (control) (scale bar: 200 μm), **h** expression of insulin gene by the parental cell line βiPLCs and FSβiPLCs treated at 1, 20 and 50 μM GCV for 7 days as determined by RT-qPCR and normalized to GAPDH relative to insulinoma (MIN6) cell line as the positive control. Astrics denotes statistical significance (**p-value < 0.01, ***p-value < 0.001)

The βiPLCs were able to form a stable cell line, amenable for subcloning from a single cell, making it suitable for genome editing, like CRISPR/Cas9-assisted knock-ins. To introduce the FailSafe modification into the cells, we used the same target vector that was designed and utilized for introducing an HSV-TK negative selectable gene into the Cdk1 gene in our previous study [26]. This modification allowed the selective elimination of dividing cells in vitro and in vivo by the HSV-TK prodrug, ganciclovir (GCV) while ensuring the survival of the non-dividing, resistant cells. We successfully produced a FailSafe Cdk1-TK knock-in βiPLC line, which we designated as FSβiPLC (Fig. 2a) [see Additional file 10].

Next, we tested the efficiency of GCV in ablating FSβiPLCs in vitro, comparing it to the killing of mouse embryonic stem cells edited with the FailSafe system (FSmES) [26]. Consistent with published data, ES cells were effectively eliminated in vitro at a GCV concentration of 1 µM (Fig. 2d) in a one-phase decay response manner. On the other hand, we observed a two-phase decay response of FSβiPLCs to GCV. The prodrug rapidly killed the FSβiPLCs during the first 4 days (first phase). Subsequently, the killing appeared to plateau (second phase) (Fig. 2b, c, d), suggesting the potential existence of a group of cells within the FSβiPLC line that either do not divide or have a slow rate of division. To further confirm the presence of slow- or non-dividing cells within the FSβiPLC population, we examined the ratio of actively dividing cells during GCV treatment at three different concentrations using immunohistochemistry for Ki67 (Fig. 2e) [see Additional file 11a–d] and conducted a comparative flow cytometry analysis (p < 0.05) for Ki67 expression (Fig. 2f**)**. Our results revealed that the GCV resistant cells were in the G0-G1 phase. Notably, while GCV treatment significantly reduced the number of cells, upon GCV withdrawal there was no increase in cell population over the next 8 days. [see Additional file 11e]. We also assessed insulin expression in cells before and after GCV treatment. As expected from beta cell development [46], our findings showed that insulin expression levels were higher in resistant cells compared to the untreated population containing actively dividing cells (Fig. 2g, h).

### Microfluidic system to study cell’s environmental response in real-time

**Fig. 3:**
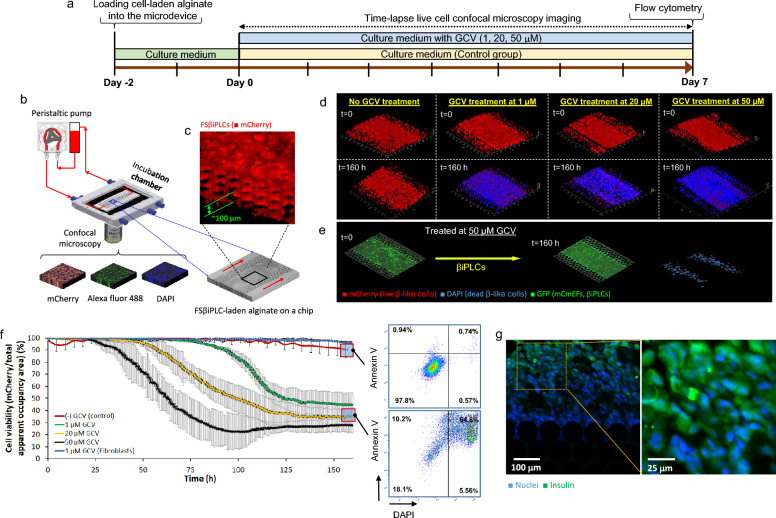
**a** Schematic diagram of the experimental procedure and **b** set-up for assessing GCV-mediated ablation of FSβiPLCs in a 3D microenvironment on-a-chip, **c** visualization of the 3D alginate-embedded FSβiPLCs in the microdevice acquired from confocal microscopy imaging, **d** confocal microscopy images show the dead (DAPI) and living FSβiPLCs (mCherry) and **e** βiPLCs (GFP) (control) in the 3D microenvironment on-a-chip at the initial (t = 0) and endpoint (t = 160 h) of GCV treatment for different GCV concentrations, **f** quantitative evaluation of changes and standard deviation (SD) in the apparent cell viability index of FSβiPLCs compared to non-treated FSβiPLCs (control) with time during 7 days of GCV treatment at different GCV concentrations, and represent results from flow cytometry, indicating the live, apoptotic and necrotic cell populations corresponding to the endpoint GCV treatment of the FSβiPLCs on-on-chip at 0 and 20 μM GCV, **g** in-situ immunohistochemistry of the FSβiPLCs embedded in alginate on-a-chip demonstrates insulin expression by some of FSβiPLCs after 7 days of GCV treatment at 20 μM GCV

As islets are among the most highly vascularized organs in the body, we have developed a transcapillary microfluidic system [see Additional file 4] to mimic a more in vivo-relevant condition. This system was used to study the transport of bioactive molecules from vessel-mimicking microchannels positioned on the sides of a central microchamber containing FSβiPLCs embedded in alginate (Fig. 3b,c). We chose alginate as a 3D microenvironment due to its favourable properties, including its hydrogel formation under physiological conditions, its gentle gel lysis for cell retrieval, its transparency for live-cell imaging, its non-animal origin and its porous network that allows nutrient/waste and bioactive molecule diffusion. By coupling this system with confocal microscopy, we were able to observe cellular dynamics during various GCV treatments in real-time. To validate the system, we employed fluorescent sodium salt (FSS) tracking substance to demonstrate that the microdevice could mimic the diffusion process of substances from microcapillaries into the 3D extracellular matrix [see Additional file 7].

We initially used the device to test the cell effectiveness of the FailSafe system’s cell ablation when the FSβiPLCs were placed in alginate in the central chamber, and GCV was diffused from the micro capillaries (Fig. 3d). Without GCV, FSβiPLCs remained viable over 160 h. However, when GCV was introduced at concentrations of 1, 20, and 50 μM, cell death occurred (Fig. 3d). To better understand how FSβiPLCs responded to GCV, we utilized time-resolved fluorescent video microscopy. This enabled us to quantitatively assess how cells reacted to varying concentrations of GCV at 1, 20, and 50 μM [see Additional files 12–16]. The FSβiPLCs showed a significant decrease (*p* < 0.01) in cell viability over time when exposed to GCV (Fig. 3f). We verified the average cell viability and insulin expression at the endpoint of GCV treatment for 160 hs using flow cytometry analysis (Fig. 3f) and in-situ immunostaining (Fig. 3g). The parental, βiPLCs (not having the HSV-TK transgene) remained viable even at the highest concentration of GCV, showing that the drug does not have non-specific cellular toxicity (viability > 96%, Fig. 3e,f).

### Evaluating the functionality of FSβiPLCs

Next, we have developed a perfusion GSIS assay on a chip to gather real-time data on glucose responsiveness dynamics of FSβiPLCs. The chip is equipped with a three-way switchable inlet that allows us to switch between low- and high-glucose Krebs–Ringer Bicarbonate (KRB) solutions in the microchannels seeded with FSβiPLCs [see Additional file 8].

To optimize the perfusion GSIS assay on a chip, we needed to determine how long it takes for the system to reach a steady state when switching from low to high glucose KRB solution. To find the best time for sampling from our microdevice, we first used computational modelling to analyse the spatiotemporal changes in insulin concentration across the device. We then validated our model by comparing the predicted values with experimental data [see Additional file 8].

**Fig. 4:**
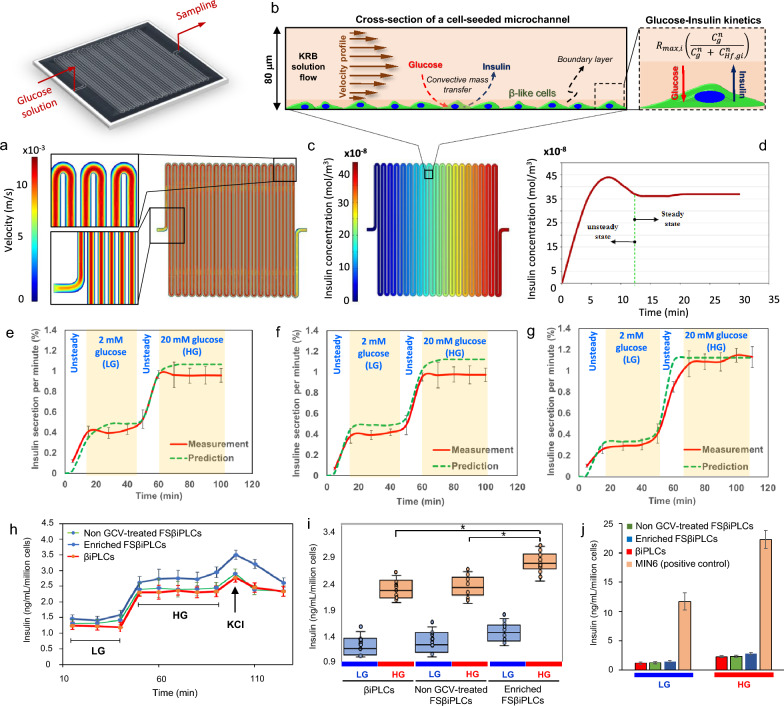
**a** Computational simulation of dynamic perfusion GSIS on-a-chip at a flow rate of 10 μL/min shows the velocity field profile, **b** a mechanistic model for the glucose transportation from the KRB flow in the microchannels into the boundary layer where glucose-uptake and insulin-secretion by the cells take place across the microfluidic device, **c** a reprehensive computational simulation of insulin concentration distribution across the microfluidic device corresponding to the KRB velocity field at the flow rate of 10 μL/min, **d** the predict values of temporal changes of insulin concentration at the sampling port, and the anticipated time for the system to reach steady state for sampling, **e**–**g** a comparison between predicted values from the computational model and experimental data of insulin secretion in terms of normalized insulin secretion per min (%) over time for the GSIS analysis of **e** βiPLCs, **f** FSβiPLCs and **g** GCV treated FSβiPLCs, **h** measured values of time-dependent insulin content per million of cells for βiPLCs (red) (control), non GCV-treated FSβiPLCs (green) and GCV-treated FSβiPLCs, **i** the statistical comparison of cumulative insulin content between βiPLCs, FSβiPLCs and the GCV-treated FSβiPLCs at LG (2 mM glucose) and HG (20 mM glucose) where asterisks denote statistically significant differences at any given data point: *p < 0.05, and **j** the comparison of insulin content between βiPLCs, FSβiPLCs, the GCV-treated FSβiPLCs and MIN6 as a positive control at LG (2 mM) and HG (20 mM)

The simulation demonstrated that there was a parabolic velocity profile across the microchannel (Fig. 4a). The fluid flow near the base of the microchannel was slow, creating a boundary layer over the seeded cells (Fig. 4b). The computational model indicated that as the KRB solution passed through the microchannel, glucose moved from the bulk flow to the boundary layer. The cells absorbed the glucose and released insulin, which was then transferred back into the bulk flow (Fig. 4b). The simulation also revealed that insulin accumulated as the KRB solution flowed towards the sampling point downstream (Fig. 4c).

Furthermore, the model has predicted fluctuations in insulin concentration at the point of sampling in response to changes in glucose concentration at the inlet. According to the model, it will take around 12 min for the system to reach a steady state, after which the fluctuations will stop (Fig. 4d). Therefore, as a necessary step before sampling when transitioning from lower to higher glucose KRB solutions, we included a waiting time of at least 12 min into our protocol. Subsequently, in order to validate the predictability of our model, we evaluated the determination of coefficients (R^2^) and compared the predicted values from our GSIS assay on a chip to the experimental data (Fig. 4e–g) [see Additional file 8].

As per the model's prediction (R^2^ > 0.95), we observed that during the unsteady state period, the insulin content in the KRB solution at the microdevice outlet gradually increased until it reached a stable level at low glucose (LG) conditions (2 mM) after approximately 12 min (Fig. 4e–g). Subsequently, we transitioned the KRB solution from LG to high glucose (HG) conditions which led to a second unsteady state, lasting for about 15 min (Fig. 4e–g).

Then, we compared the insulin secretion levels of three cell lines βiPLCs, non-GCV-treated FSβiPLCs, and non-dividing cell-enriched (GCV treated) FSβiPLCs. Our results demonstrated that all three cell lines responded to glucose and KCl by secreting insulin (Fig. 4h, i). The glucose-responsive insulin secretion profile of βiPLCs and no-GCV treated FSβiPLCs were not significantly different at LG and HG, and both had similar insulin content when depolarized by KCl (Fig. 4h). However, the GCV treated non-dividing cell-enriched FSβiPLCs had significantly higher insulin secretion (*p* < 0.05) at HG and insulin content at depolarization than the other two groups (Fig. 4h, i).

Lastly, we compared the insulin content of the three lines βiPLCs, non-GCV-treated FSβiPLCs, and non-dividing cell-enriched (GCV treated) FSβiPLCs, against a positive control. Although primary mouse islets would be ideal for comparison, their size poses a challenge for our perfusion GSIS assay-on-chip, as they can clog the microchannels. As an alternative, we used the MIN6 insulinoma cell line (Sigma-Aldrich, SCC623), which retains key characteristics of mature mouse beta cells. The results showed that the insulin content of three cell lines was lower than that of MIN6 cells under both LG and HG conditions (Fig. 4j).

## Discussion

The emergence of human embryonic stem cells in 1998 [47] has brought unprecedented opportunities to regenerative medicine. These pluripotent cells have the unique ability to differentiate into any cell type in the body, thus making them a promising source for generating replacement cells for various diseases. Eight years later, the discovery of reprogramming somatic cells to pluripotency [48, 49] has further advanced the potential of cell replacement therapy for addressing the ever-expanding spectrum of degenerative diseases. Moreover, reprogramming with specific transcription factors has indicated the potential for changing cell stages both across [50] and along developmental lineages [51]. Our earlier research has demonstrated the feasibility of converting fully differentiated, non-dividing alveolar type II cells of the lung into induced progenitor-like cells (iPLCs) through interrupted reprogramming while preserving the cells’ commitment to the lung lineage [21]. Although these cells ceased proliferation upon turning off the reprogramming transgenes, they successfully differentiated into multiple lung cell types in vitro and in vivo following orthotopic transplantation [21].

Here, we report the generation of a permanent cell line by interrupted reprogramming of the mouse pancreas's beta cells. The interruption was timed to shortly before the cells stopped producing insulin. The resulting cells retained the crucial characteristic of beta cells, which is their ability to secrete insulin in response to glucose. To demonstrate that the cells enable genome editing and ensure safety if transplanted, we introduced our highly reliable FailSafe kill switch [26] into subclones and selected one for further characterization. Remarkably, we found that even after subcloning and extensive expansion, the cell population remained heterogeneous, consisting of both proliferative and non-proliferative compartments. Furthermore, the non-proliferative compartment responded significantly (p < 0.05) better to high glucose regarding insulin secretion.

The implementation of the FailSafe system serves two purposes; to protect against any uncontrolled growth in case the cells are transplanted into recipients in a T1D mouse model and to enrich for the therapeutically superior non-proliferative compartment before transplantation. We have already demonstrated the usefulness of both utilizations of the FailSafe system; in the original publication [26] and in a more recent publication targeting Parkinson’s disease in a mouse model [27], respectively.

This publication focuses primarily on the importance of developing informative in vitro assays to test a novel cell line’s properties before conducting tests in animal models. It is important to note that while this study demonstrates the generation of a mouse cell line with glucose-responsive insulin-secreting properties and equipped with a FailSafe kill-switch, there are several limitations to consider for extrapolating these findings for potential translation in human studies. The interrupted reprogramming of mouse pancreatic beta cells in this study relies on a transgenic mouse model that ensures controlled expression of reprogramming factors only in pancreatic beta cells. For translation to human studies, a significant challenge lies in designing a human cell-specific method to deliver reprogramming factors only to human beta cells in a controlled manner. Additionally, because the mouse insulin promoter (MIP) and the human insulin promoter differ in their regulatory elements, human βiPLCs may differ in retaining critical properties of their cell origin, most importantly glucose-responsive insulin secretion, after partial reprogramming, compared to their mouse counterparts. Furthermore, our previous study [26] demonstrated the reliability and effectiveness of the FailSafe kill-switch system in human stem cells. For future clinical applications, we plan to use this stringent FailSafe system in the human stem cell-derived insulin secreting cells, providing a robust safeguard against uncontrolled proliferation.

To this end, we have developed two microfluidic systems, a perfusion GSIS assay on a chip and a transcapillary microfluidic system. The latter system is designed to mimic the transportation of biologically active molecules from side-channels to the central chamber containing the FSβiPLCs in a 3D microenvironment. The results from the perfusion GSIS-on-a-chip demonstrated that the cells functionally secrete insulin in response to glucose over time rather than in a single burst due to changes in osmolarity. The dynamics of insulin secretion by the cells over time cannot be captured by the standard static assay. Additionally, the transcapillary microfluidic system provided insights into the dynamics of the FailSafe kill-switch system's functionality in cells in response to a diffusional pro-drug within a perfused 3D microenvironment. These organ-on-chip systems provided us with important information about the cells, emphasizing the usefulness of such a system to characterize the basic properties of the novel cell type equipped with the FailSafe kill-switch system with the potential to generate therapeutic cells for targeting T1D.

## Conclusions

Our perfusion GSIS assay-on-chip and transcapillary microfluidic system provided valuable tools for obtaining more predictive insights into a novel glucose-responsive insulin-secreting cell line with a FailSafe kill-switch. The cells demonstrated functional insulin secretion in response to changes in glucose concentration. The FailSafe system effectively eliminated dividing cells while the resistant non-dividing cells exhibited higher levels of insulin secretion compared to their proliferating counterparts. It suggests a potential implementation of the FailSafe kill-switch to isolate the preferred compartment, non-dividing cells, for cell replacement therapy in the treatment of T1D.

## Supplementary Information

Additional file 1. Supplementary Figure: Screening 85 clones for Ins expression, and doxycycline-induced reprograming of pancreatic islets of the quadruplet transgenic mice in vitro. (a) Insulin expression level determined by qPCR for 85 lines was used for selecting the top three lines for subsequent GSIS analysis. (b) The transgene#4 (MIP-EGFP) in the quadruplet transgenic mice enables visualization of beta cells over tide using confocal microscopy. In the presence of dox, the transgene#3 enables OKMS expression by the beta cells in the islets represented by mCherry signal.

Additional file 2. Supplementary Video: Monitoring the reprograming of pancreatic islets of the quadruplet transgenic mice in vitro. The representative confocal fluorescent video microscopy of dox-inducible reprogramed MIP-GFP mouse pancreatic islets in the presence of dox shows that mice pancreatic β cells (GFP) undergo cell reprograming in the presence of dox that results in OKMS expression (mCherry) which progressively increases with time.

Additional file 3. Supplementary Video: Monitoring the reprograming of pancreatic islets of the quadruplet transgenic mice in vitro. The representative confocal fluorescent video microscopy of dox-inducible reprogramed MIP-GFP mouse pancreatic islets in the absence of dox shows that mice pancreatic β cells (GFP) do not express OKMS (mCherry).

Additional file 4. Supplementary Figure: A transcapillary-resembling microfluidic system development, specifications and integration with confocal microscopy. The microdevice structure mimics the capillary convection and transcapillary diffusion of bioactive molecules into a central 3D microenvironment. It consists of micropillar PDMS arrays (100 μm high, 10 μm apart) that separate two side microchannels (450 µm wide) from a central microchamber (550 µm wide) where culture medium flows through the side channels from which nutrients and GCV are transported into the 3D microenvironment in the central microchamber. The experimental setup consisting of a peristaltic pump that injects the culture medium with DAPI or FSS from the reservoir into the microfluidic device. The microfluidic device is placed in the incubation chamber (37 °C, 5% CO2). The time-lapse fluorescent imaging is performed using a two-photon confocal microscope (LSM750, Zeiss, Germany).

Additional file 5. Supplementary Methods: Additional information providing more details for Materials and Methods.

Additional file 6. Supplementary Figure: Fabrication process to create microfluidic devices using soft lithography. Schematic diagram of the fabrication process to create two PDMS-based microfluidic systems.

Additional file 7. Supplementary Figure: Validation of transcapillary diffusion-controlled mass transfer across the 3D microenvoronment-on-a-chip. (a) The real-time confocal fluorescence microscopy imaging of the microdevice to trace a model cue, fluorescent sodium salt solution (FSS) across the side microchannels, micropillar arrays and the 3D hydrogel in the central microchamber over 15 min at three flow rates of 5, 10 and 15 μL/min, (b, c) the normalized values of FSS concentration distribution indicates no significant difference in spatial FSS concentration gradient across the alginate-loaded microchamber between different flow rates (5, 10 and 15 μL/min) at different time points.

Additional file 8. Supplementary Figure: The perfusion glucose stimulated insulin secretion (GSIS) assay-on-a-chip. (a) The design and specifications of the microfluidic-based perfusion GSIS on-a-chip consisting of a 1.5 m long and 800 µm wide microchannel with switchable inlets corresponding to LG, HG and KCl+HG solutions. Using phase contrast imaging, 8 different locations of cell-seeded microchannels were imaged and analyzed in ImageJ to estimate the cell density of adhered cells (cells per unit area of microchannel) to be used for normalizing the kinetic data of insulin secretion. (b) The initial and boundary conditions of the perfusion GSIS-on-a-chip was applied for CFD modeling to predict insulin secretion distribution across the microchannels over time. The predictability of the model was assessed by evaluating the coefficient of determination between predicted values from the CFD model and measured values of insulin secretion obtained from the perfusion GSIS assay on-chip corresponding to (c) βiPLCs, (d) FSβiPLCs and (e) GCV treated FSβiPLCs.

Additional file 9. Supplementary Table: The list of primers used for RT-qPCR and genomic PCR.

Additional file 10. Supplementary Figure: Generation of FSβiPLCs through FailSafe system integration into βiPLCs. (a) Strategy for identifying candidate targeted clones using Puromycin resistant gene and mCherry reporter. (b) Isolation of candidate FSβiPLCs ussing FACS based on high mCherry expression from the Cdk1 knock-in vector. (c) Genomic PCR confirms presence of wild type allele in passages #35 and #39 of selected clones. (d) PCR verifies correct 5’ and 3’ insertion of FailSafe vector in the Cdk1 targeted allele.

Additional file 11. Supplementary Figure: Phase contrast and fluorescence images depict FSβiPLCs stained with DAPI (blue) and anti-Ki67 (green) at days 0, 4, and 7 of GCV treatment across three concentrations (a) 1 µM, (b) 20 µM, and (c) 50 µM (scale bar: 50 μm). Decreasing Ki67 expression over time indicates reduced cell proliferation, suggesting that the majority of surviving FSβiPLCs become non-proliferative after 7 days of GCV treatment. (d) The quantitative flow cytometry shows a significant decrease (p<0.05) in Ki67 expression by FSβiPLCs after 7 days of GCV treatment for different GCV concentrations. (e) Withdrawal of GCV (1 μM) at day 7 did not result in cell population increase over 8 days.

Additional file 12. Supplementary Video: Confocal video microscopy-based assessment of GCV sensitivity of FSβiPLCs within a 3D microenvironment on-a-chip. The time-lapsed confocal fluorescent imaging and quantitative analysis reveal no significant changes in live (mCherry) and dead (DAPI) cell populations over time, indicating a high cell viability index over time in the absence of GCV.

Additional file 13. Supplementary Video: Confocal video microscopy-based assessment of GCV sensitivity of FSβiPLCs within a 3D microenvironment on-a-chip at 1 μM GCV. Time-lapsed confocal fluorescent imaging and quantitative analysis reveal fluctuations in live (mCherry) and dead (DAPI) cell populations over time, indicating a decline in cell viability index at 1 μM GCV.

Additional file 14. Supplementary Video: Confocal video microscopy-based assessment of GCV sensitivity of FSβiPLCs within a 3D microenvironment on-a-chip at 20 μM GCV. Time-lapsed confocal fluorescent imaging and quantitative analysis reveal fluctuations in live (mCherry) and dead (DAPI) cell populations over time, indicating a decline in cell viability index at 20 μM GCV.

Additional file 15. Supplementary Video: Confocal video microscopy-based assessment of GCV sensitivity of FSβiPLCs within a 3D microenvironment on-a-chip at 50 μM GCV. Time-lapsed confocal fluorescent imaging and quantitative analysis reveal fluctuations in live (mCherry) and dead (DAPI) cell populations over time, indicating an earlier decline in cell viability index at 50 μM GCV.

Additional file 16. Supplementary Video: A comparison of FSβiPLCs sensitivity between different GCV concentrations within a 3D microenvironment on-a-chip. A real-time comparison of GCV-mediated ablation efficacy in FSβiPLCs within a microenvironment-on-a-chip across various concentrations (1, 20, and 50 μM) compared to the control group. Dead cells are identified by DAPI staining of their nuclei.

## Data Availability

All data generated, analysed and reported during this study are included in this published article and its supplementary information files.

## Abbreviations

BSA: Bovine serum albumin
βiPLCs: β cell-induced progenitor-like cells
CFD: Computational fluid dynamics
CPS: Commitment point to induced pluripotent stem
Cre: Cyclization
Dox: Doxycycline
FACS: Fluorescence-activated cell sorting
FSS: Fluorescent sodium salt
FSβiPLCs: FailSafe β cell-induced progenitor-like cells
FSmESC: FailSafe mouse embryonic stem cell
GCV: Ganciclovir
GSIS: Glucose-stimulated insulin secretion
hPSCs: Human pluripotent cells
HG: High glucose
HSV: Herpes simplex virus
iPLCs: Induced lung progenitor-like cells
iPS: Induced pluripotent stem
KRB: Krebs-Ringer Bicarbonate
LG: Low glucose
MEM: Minimum essential medium
MIP: Mouse insulin promoter
OKMS: Oct4, Klf4, c-Myc, and Sox2
PBS: Phosphate-buffered saline
PCR: Polymerase chain reaction
PFA: Paraformaldehyde
PNR: Point-of-no-return
T1D: Type 1 diabetes
TK: Thymidine kinase
