# Supplementary figures and images for: Microfluidic chip systems for characterizing glucose-responsive insulin-secreting cells equipped with FailSafe kill-switch
Source: Stem Cell Res Ther. 2024 Dec 18;15:486. doi: 10.1186/s13287-024-04059-7 (PMC11656860; doi:10.1186/s13287-024-04059-7)

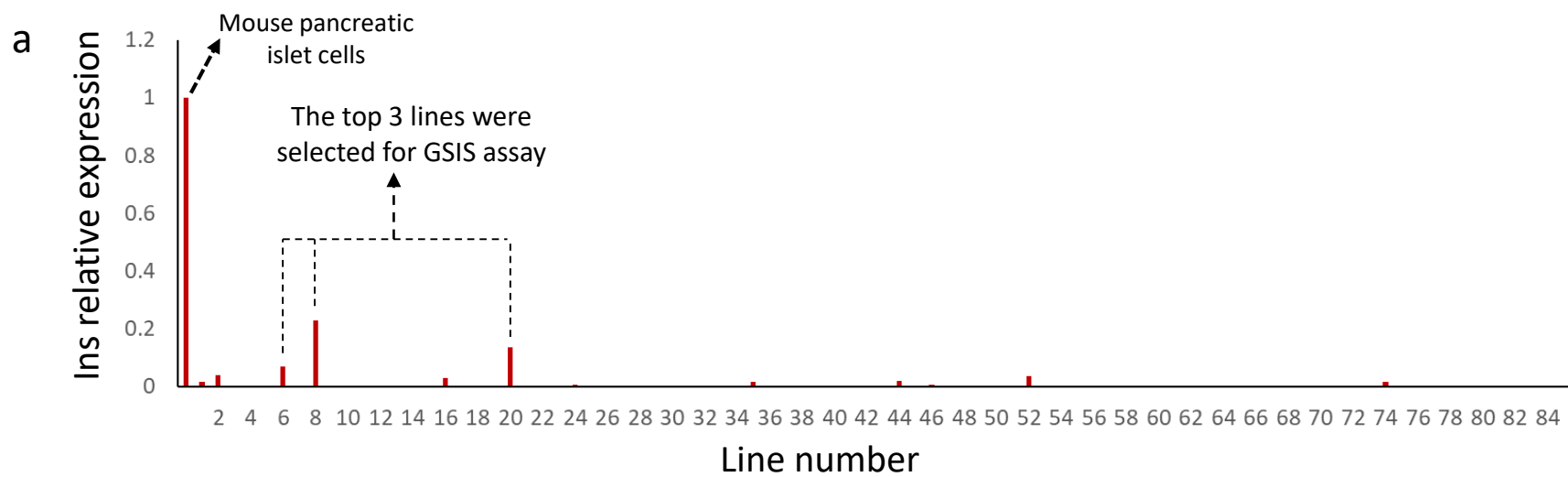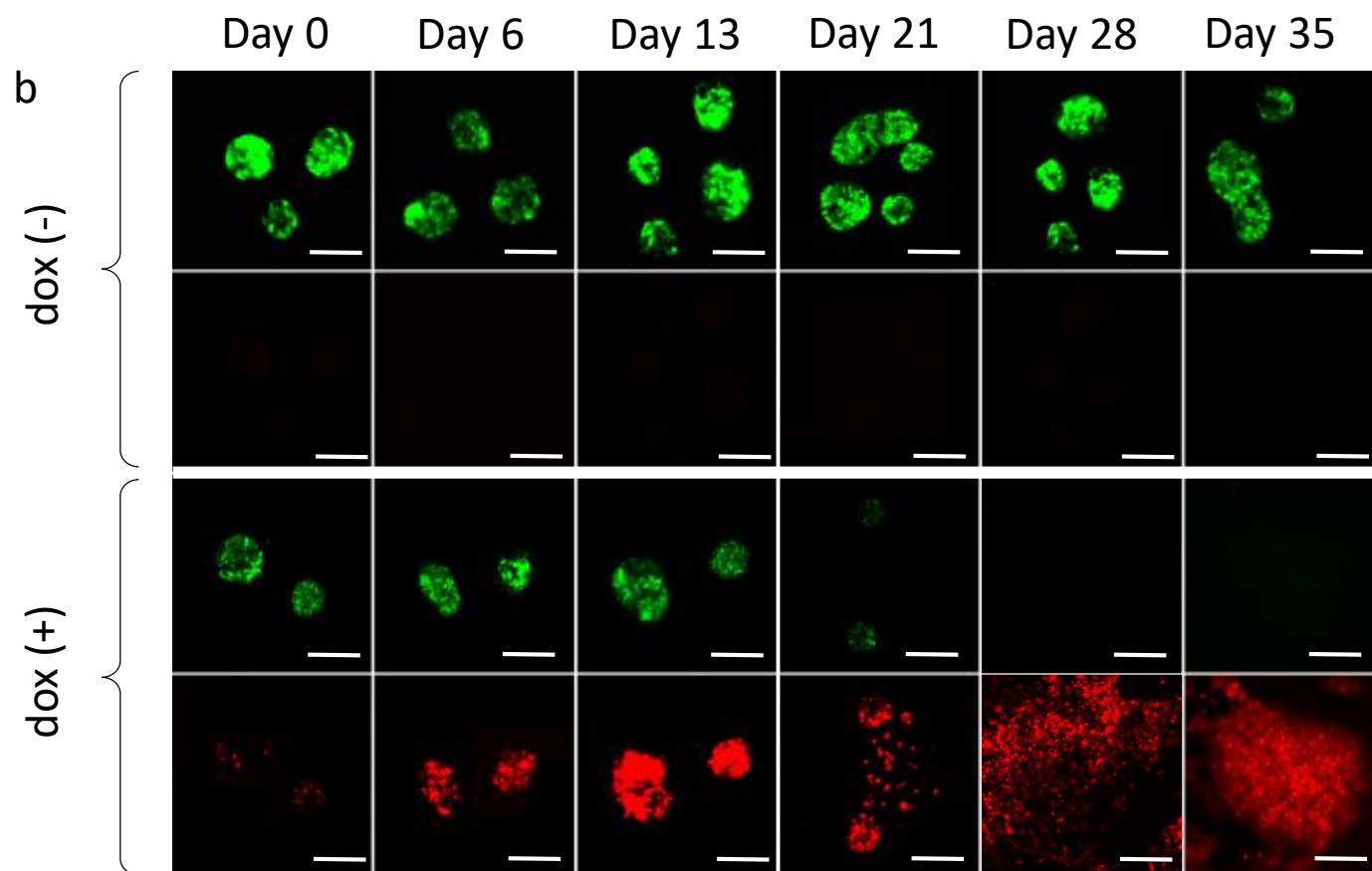

Supplement: Supplementary file 1 — Additional file 1. Supplementary Figure: Screening 85 clones for Ins expression, and doxycycline-induced reprograming of pancreatic islets of the quadruplet transgenic mice in vitro. (a) Insulin expression level determined by qPCR for 85 lines was used for selecting the top three lines for subsequent GSIS analysis. (b) The transgene#4 (MIP-EGFP) in the quadruplet transgenic mice enables visualization of beta cells over tide using confocal microscopy. In the presence of dox, the transgene#3 enables OKMS expression by the beta cells in the islets represented by mCherry signal. [file 13287_2024_4059_MOESM1_ESM.pdf]

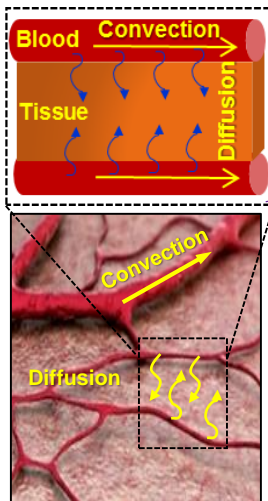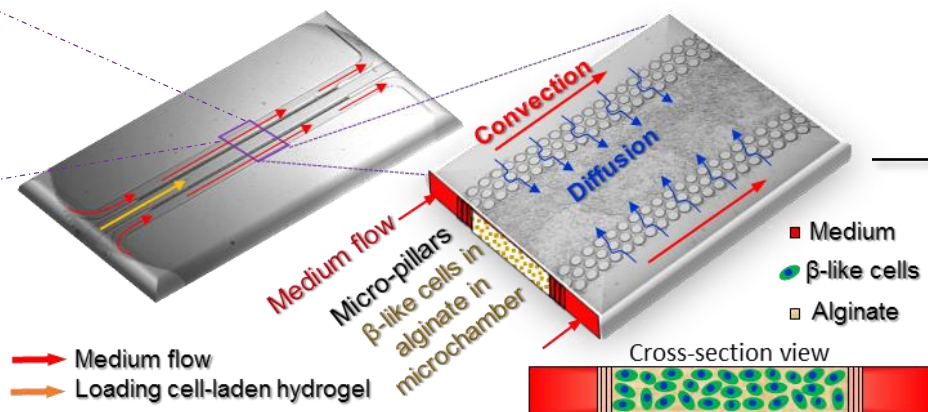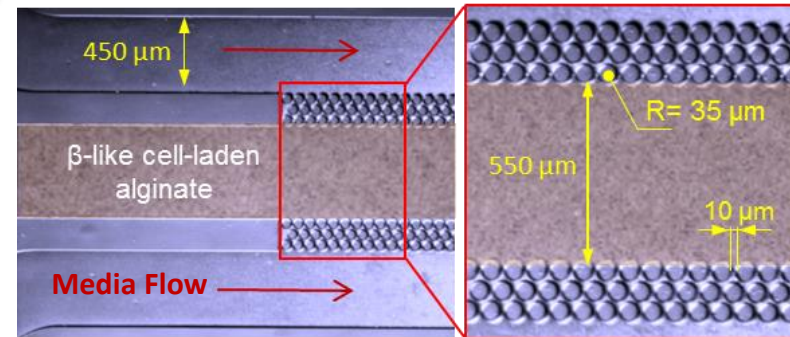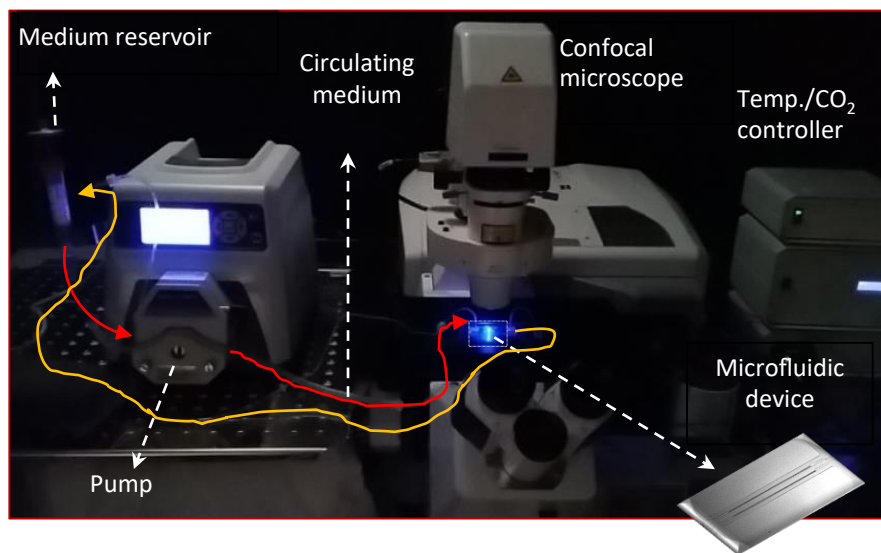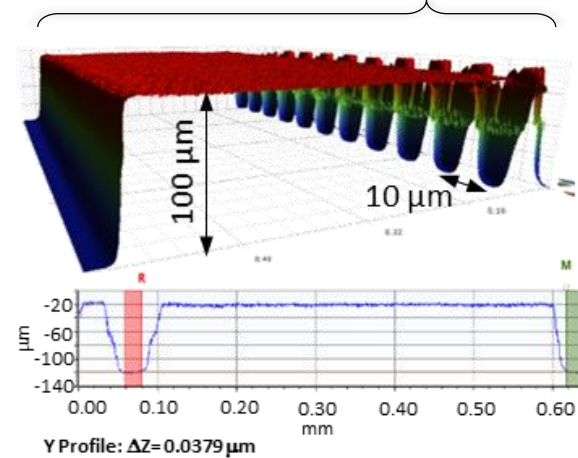

Supplement: Supplementary file 4 — Additional file 4. Supplementary Figure: A transcapillary-resembling microfluidic system development, specifications and integration with confocal microscopy. The microdevice structure mimics the capillary convection and transcapillary diffusion of bioactive molecules into a central 3D microenvironment. It consists of micropillar PDMS arrays (100 μm high, 10 μm apart) that separate two side microchannels (450 µm wide) from a central microchamber (550 µm wide) where culture medium flows through the side channels from which nutrients and GCV are transported into the 3D microenvironment in the central microchamber. The experimental setup consisting of a peristaltic pump that injects the culture medium with DAPI or FSS from the reservoir into the microfluidic device. The microfluidic device is placed in the incubation chamber (37 °C, 5% CO2). The time-lapse fluorescent imaging is performed using a two-photon confocal microscope (LSM750, Zeiss, Germany). [file 13287_2024_4059_MOESM4_ESM.pdf]

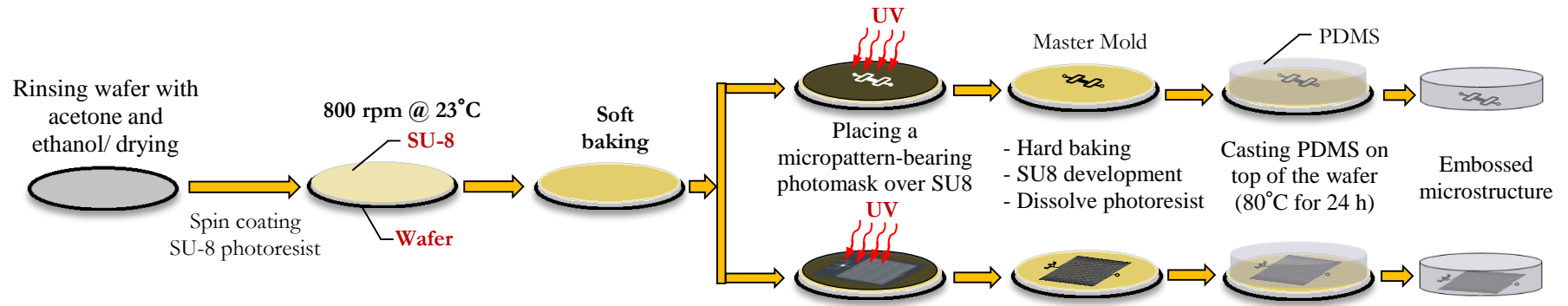

Supplement: Supplementary file 6 — Additional file 6. Supplementary Figure: Fabrication process to create microfluidic devices using soft lithography. Schematic diagram of the fabrication process to create two PDMS-based microfluidic systems. [file 13287_2024_4059_MOESM6_ESM.pdf]

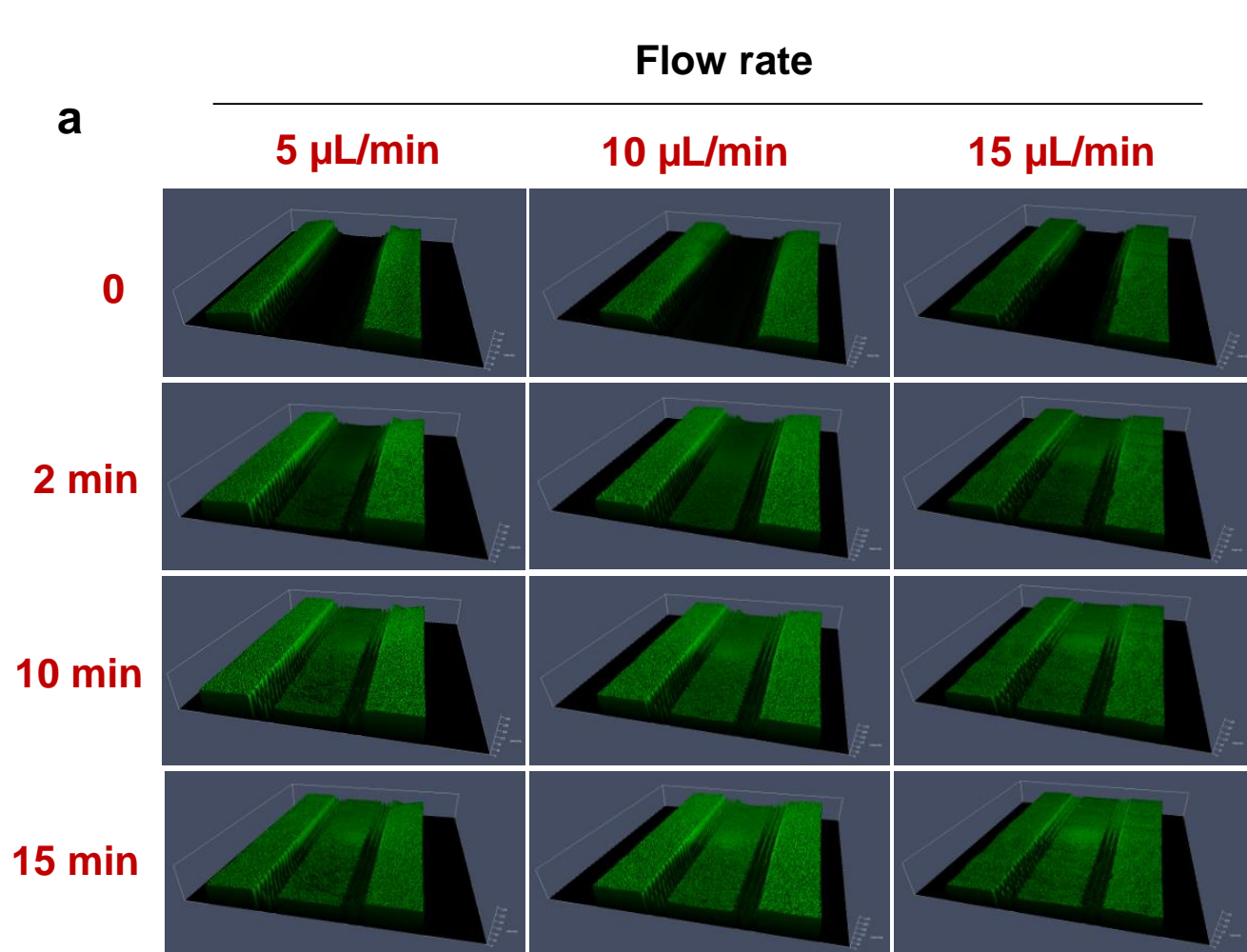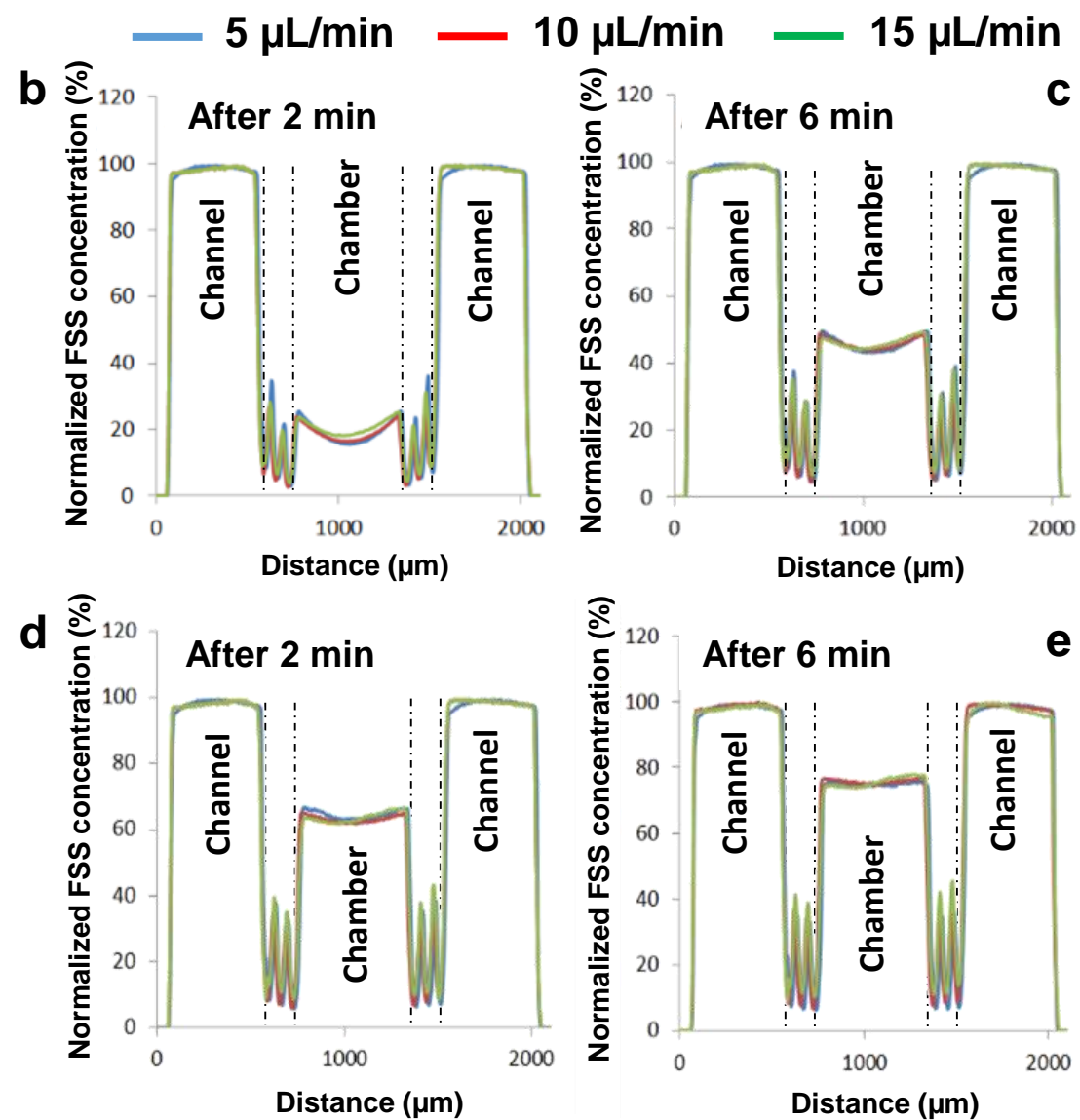

Supplement: Supplementary file 7 — Additional file 7. Supplementary Figure: Validation of transcapillary diffusion-controlled mass transfer across the 3D microenvoronment-on-a-chip. (a) The real-time confocal fluorescence microscopy imaging of the microdevice to trace a model cue, fluorescent sodium salt solution (FSS) across the side microchannels, micropillar arrays and the 3D hydrogel in the central microchamber over 15 min at three flow rates of 5, 10 and 15 μL/min, (b, c) the normalized values of FSS concentration distribution indicates no significant difference in spatial FSS concentration gradient across the alginate-loaded microchamber between different flow rates (5, 10 and 15 μL/min) at different time points. [file 13287_2024_4059_MOESM7_ESM.pdf]

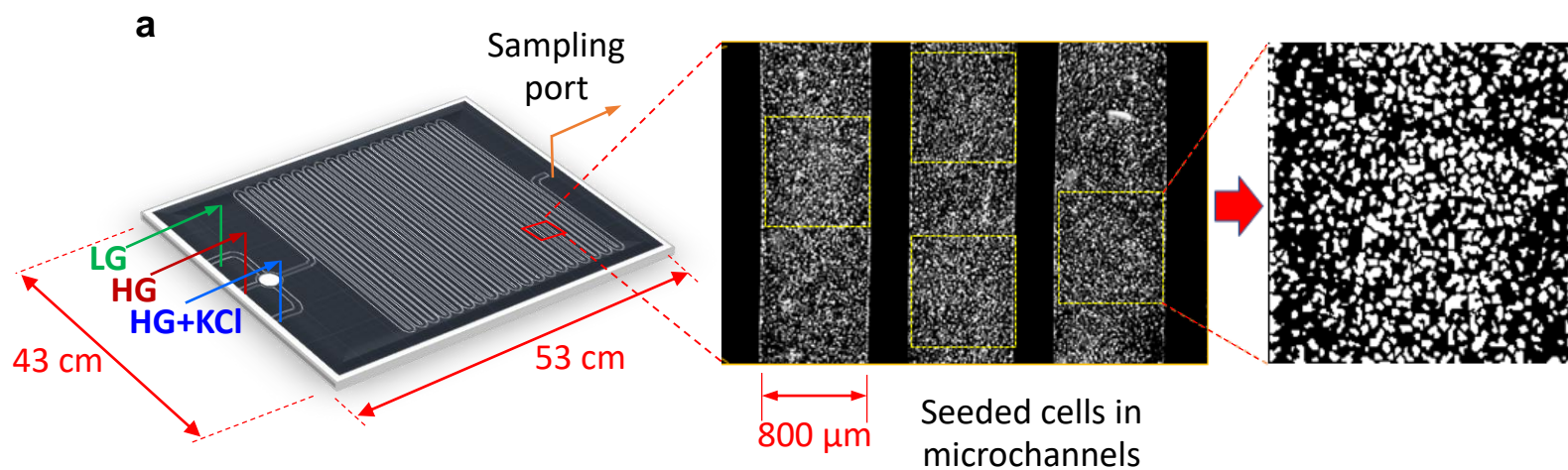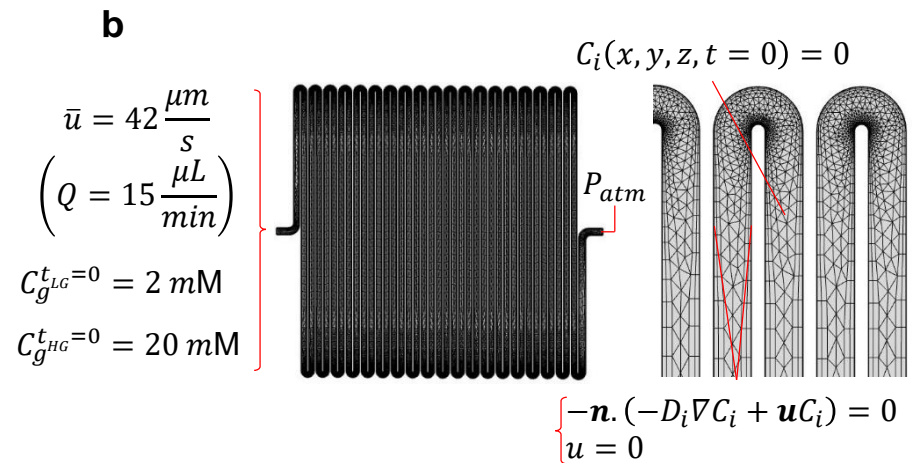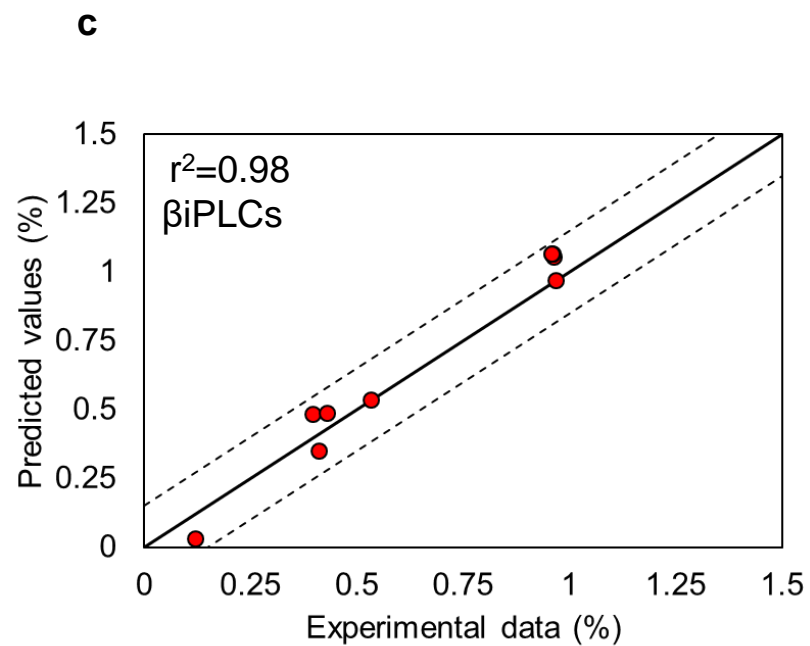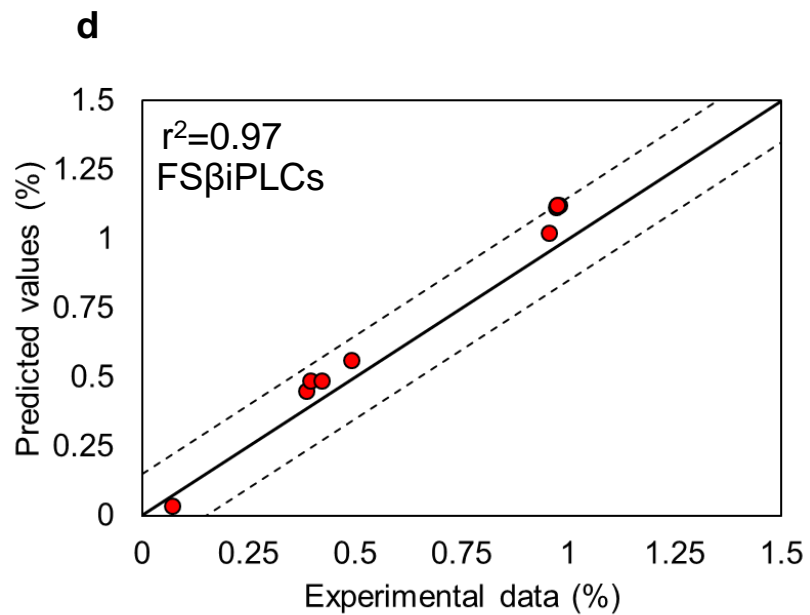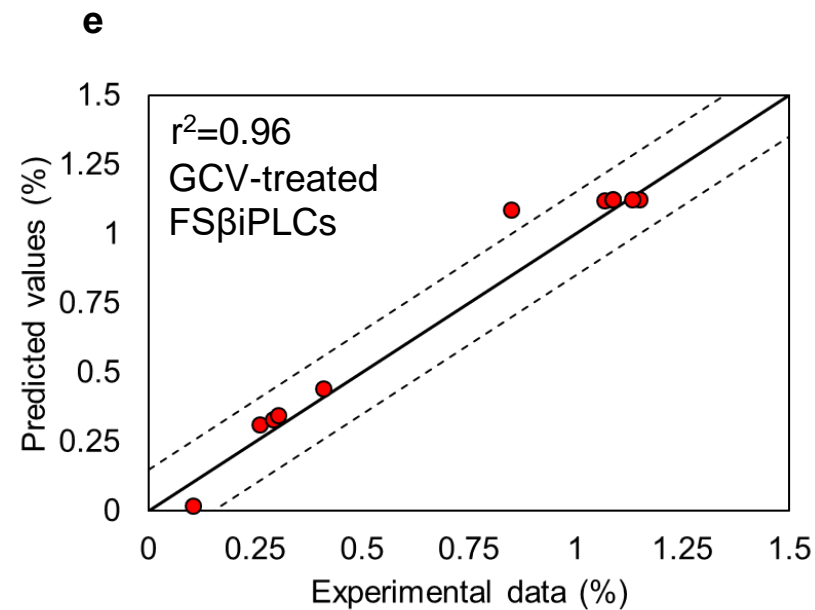

Supplement: Supplementary file 8 — Additional file 8. Supplementary Figure: The perfusion glucose stimulated insulin secretion (GSIS) assay-on-a-chip. (a) The design and specifications of the microfluidic-based perfusion GSIS on-a-chip consisting of a 1.5 m long and 800 µm wide microchannel with switchable inlets corresponding to LG, HG and KCl+HG solutions. Using phase contrast imaging, 8 different locations of cell-seeded microchannels were imaged and analyzed in ImageJ to estimate the cell density of adhered cells (cells per unit area of microchannel) to be used for normalizing the kinetic data of insulin secretion. (b) The initial and boundary conditions of the perfusion GSIS-on-a-chip was applied for CFD modeling to predict insulin secretion distribution across the microchannels over time. The predictability of the model was assessed by evaluating the coefficient of determination between predicted values from the CFD model and measured values of insulin secretion obtained from the perfusion GSIS assay on-chip corresponding to (c) βiPLCs, (d) FSβiPLCs and (e) GCV treated FSβiPLCs. [file 13287_2024_4059_MOESM8_ESM.pdf]

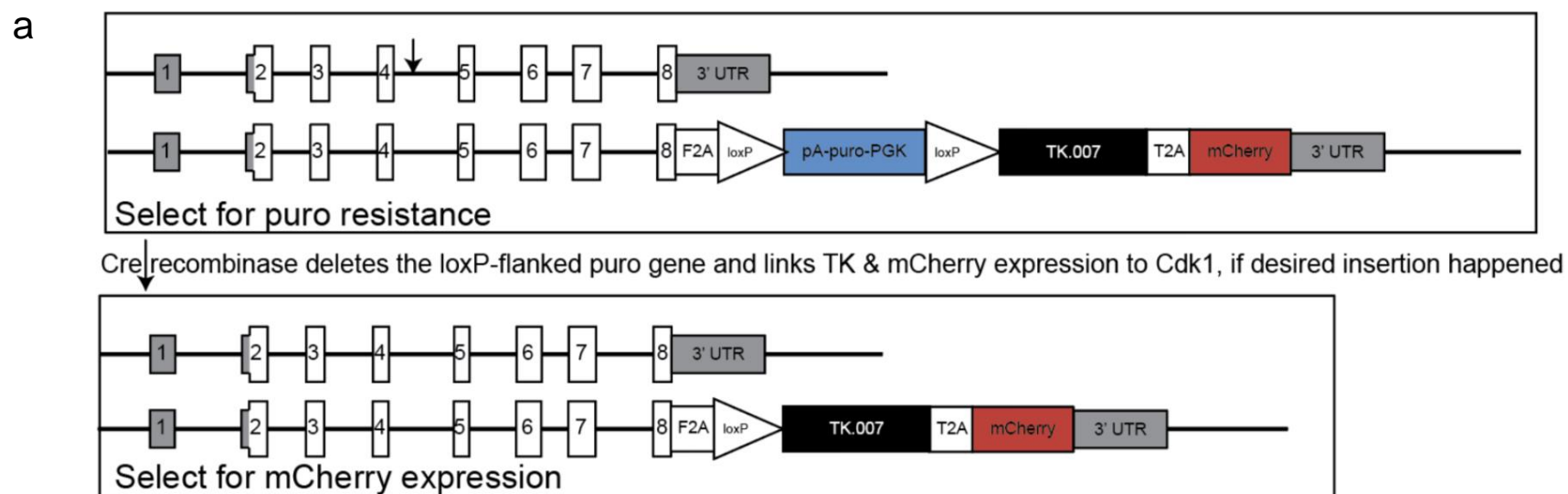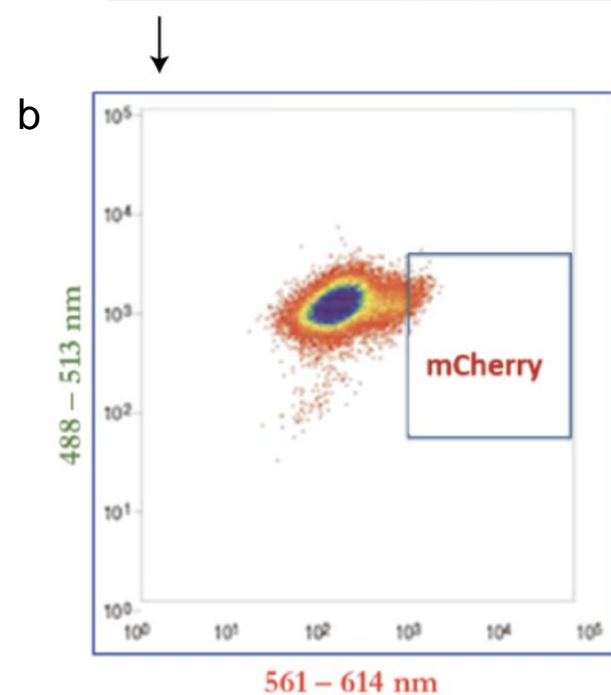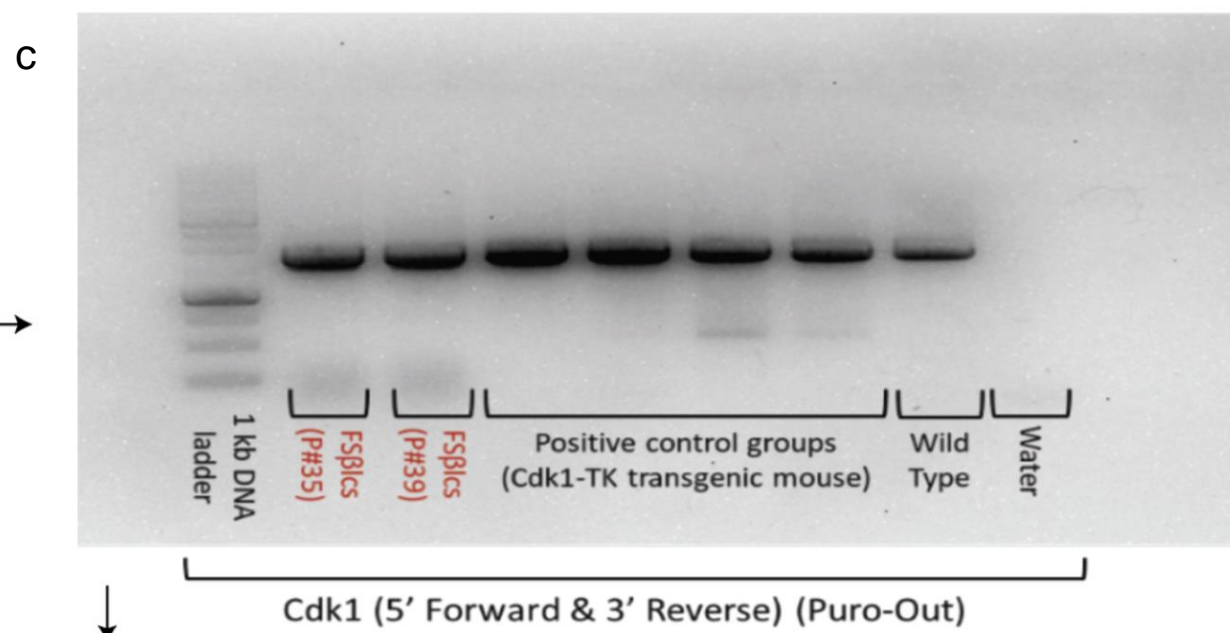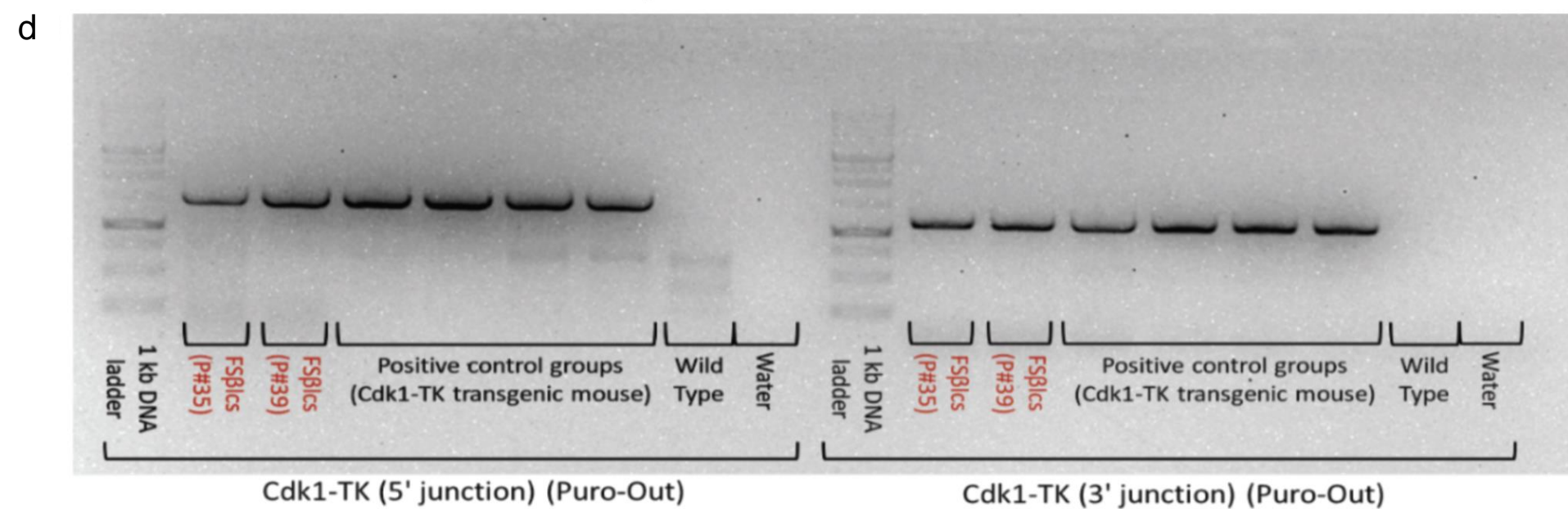

Supplement: Supplementary file 10 — Additional file 10. Supplementary Figure: Generation of FSβiPLCs through FailSafe system integration into βiPLCs. (a) Strategy for identifying candidate targeted clones using Puromycin resistant gene and mCherry reporter. (b) Isolation of candidate FSβiPLCs ussing FACS based on high mCherry expression from the Cdk1 knock-in vector. (c) Genomic PCR confirms presence of wild type allele in passages #35 and #39 of selected clones. (d) PCR verifies correct 5’ and 3’ insertion of FailSafe vector in the Cdk1 targeted allele. [file 13287_2024_4059_MOESM10_ESM.pdf]

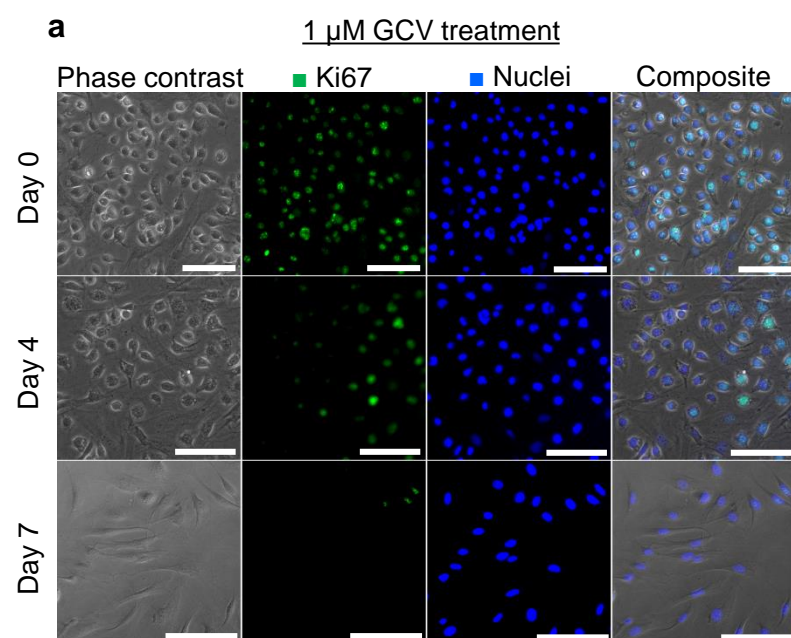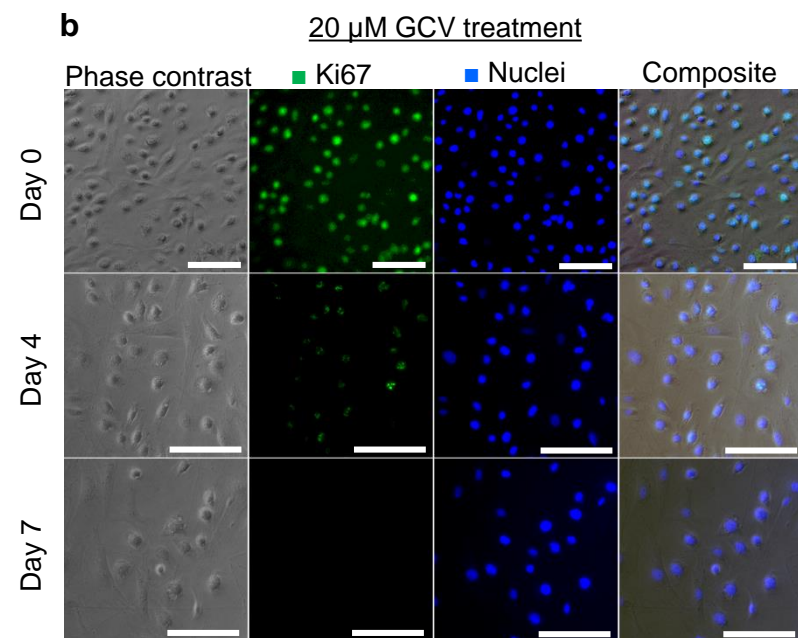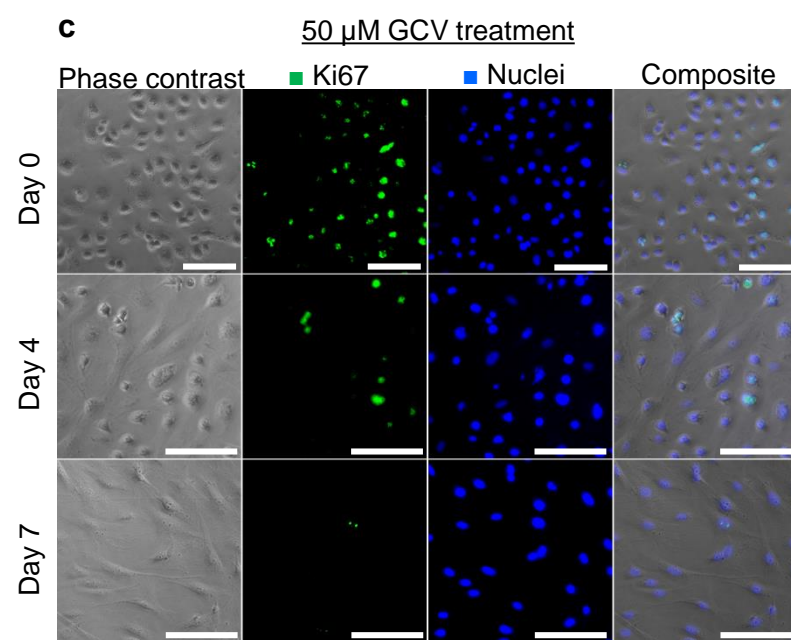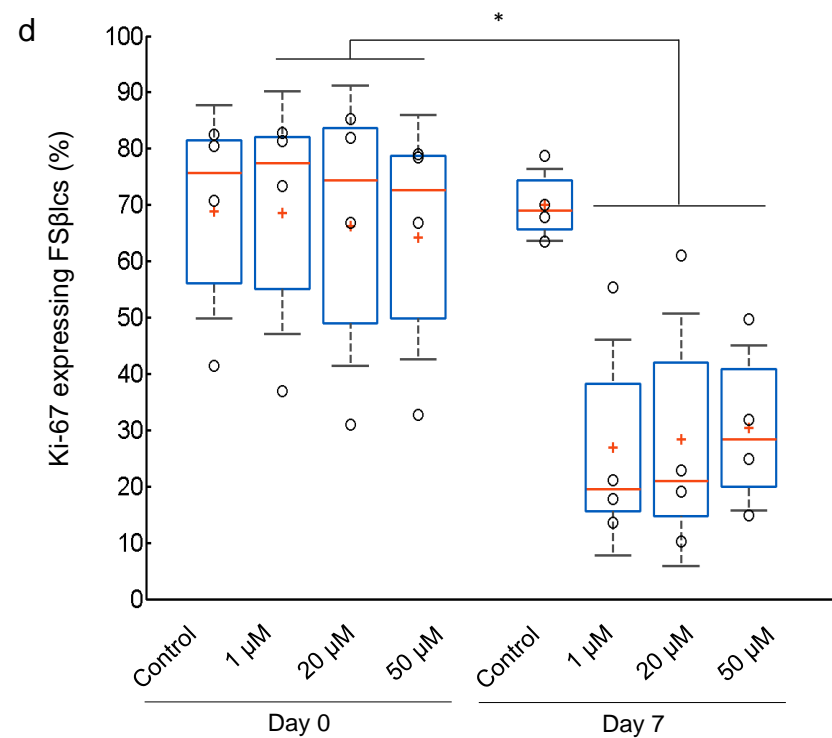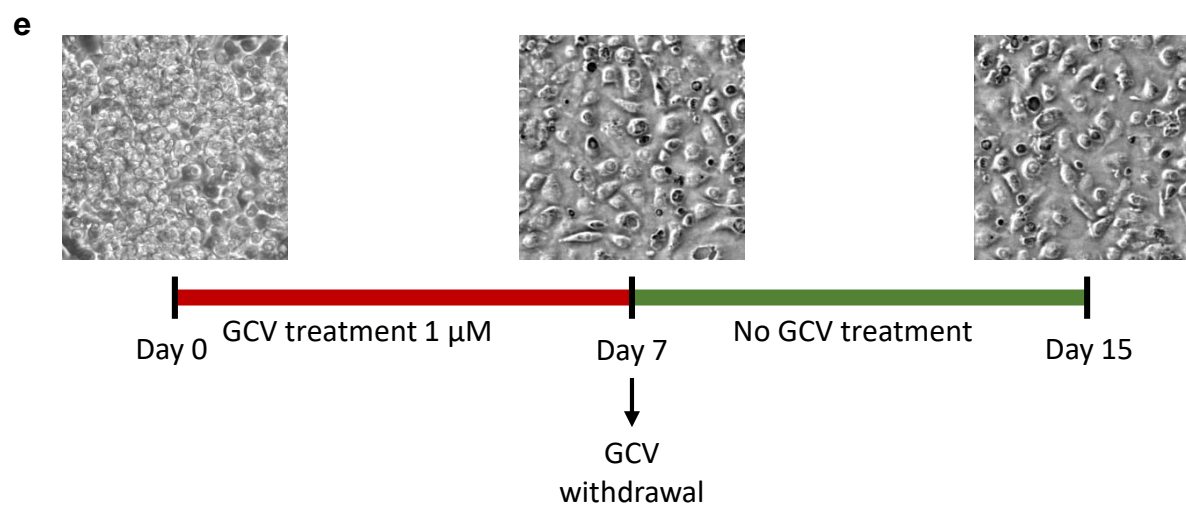

Supplement: Supplementary file 11 — Additional file 11. Supplementary Figure: Phase contrast and fluorescence images depict FSβiPLCs stained with DAPI (blue) and anti-Ki67 (green) at days 0, 4, and 7 of GCV treatment across three concentrations (a) 1 µM, (b) 20 µM, and (c) 50 µM (scale bar: 50 μm). Decreasing Ki67 expression over time indicates reduced cell proliferation, suggesting that the majority of surviving FSβiPLCs become non-proliferative after 7 days of GCV treatment. (d) The quantitative flow cytometry shows a significant decrease (p<0.05) in Ki67 expression by FSβiPLCs after 7 days of GCV treatment for different GCV concentrations. (e) Withdrawal of GCV (1 μM) at day 7 did not result in cell population increase over 8 days. [file 13287_2024_4059_MOESM11_ESM.pdf]
