# Supplementary material for: Microfluidic chip systems for characterizing glucose-responsive insulin-secreting cells equipped with FailSafe kill-switch
Source: Stem Cell Res Ther. 2024 Dec 18;15:486. doi: 10.1186/s13287-024-04059-7 (PMC11656860; doi:10.1186/s13287-024-04059-7)
Supplement: Supplementary file 5 — Additional file 5. Supplementary Methods: Additional information providing more details for Materials and Methods. [file 13287_2024_4059_MOESM5_ESM.docx]

**SUPPLEMENTARY MATERIALS**

**Development of a microfluidic-based GCV sensitivity assay in 3D microenvironment**

The microfluidic device was designed in AutoCAD software. The design consists of two side microchannels and a central microchamber which are separated by arrays of micropillars (Supplementary Fig. 2b). Then, we converted the design patterns in AutoCAD to photomasks using a laser pattern generator with an encoder resolution of 2 nm (Heidelberg Instruments Mikrotechnik, Germany) and created the microfeatures by photolithography process. Next, we spin-coated the negative photoresist (SU-8 3000) onto a silicon wafer and baked it for 10 min at 65°C and 45 min at 95 °C followed by UV exposure onto the photomask aligned with the baked photoresist (Supplementary Fig. 2a). After developing the master mold using SU8 developer, we exposed the master mold to trimethylchlorosilane vapor for 5 min. Next, we used the coated SU8 master mold and fabricated the microfluidic device using soft lithography with polydimethylsiloxane (PDMS) (Ellsworth Adhesive, Canada) (Supplementary Fig. 2a).

**Validation of diffusion-controlled mass transfer across the 3D microenvoronment-on-a-chip**

Cells were mixed with the filter-sterilized UPLVHG alginate (1.8% w/v) and then loaded into the central microchamber of the device using a 1 mL syringe. We connected the device to a peristaltic pump and positioned the device in the incubator chamber of a confocal microscope (LSM750, Zeiss, Germany) (Supplementary Fig. 2c). Before starting imaging, a traceable model cue, fluorescent sodium salt solution (FSS) (100 mM in sodium bicarbonate buffer, pH 8.3) (Millipore Sigma, Canada) was added to the media in the reservoir. The media containing FSS was injected into the side microchannels at different flow rates (5, 10 and 15 μL/min) while capturing the fluorescent signal of FSS across the microchannels and microchamber in real-time using confocal microscopy imaging (LSM750, Zeiss, Germany). Experiments were conducted in triplicate for 15 min at a constant gate and exposure time of 100 ms. For quantitative analysis, we calibrated the fluorescence readings by measuring the average fluorescent signal intensity at different FSS solution concentrations (0 – 0.01 μM). A calibration curve with the best linear fit (r^2^>0.98) was generated and used to quantify fluorescence readings. Additionally, we normalized the intensity of fluorescent signals by the maximum intensity value corresponding to the constant FSS flow in the side microchannels to scale the readings for all experiments. the normalized values of FSS concentration distribution revealed that there was no significant difference in spatial distribution of FSS concentration gradient across the 3D hydrogel in the microchamber between different flow rates (5, 10 and 15 μL/min) at different time points. It indicates that FSS diffusion is the major mechanism of mass transfer from side channels into the microchamber while the effect of hydrodynamic pressure of different flow rates has insignificant effect on FSS transportation into the central microchamber.

**Live cell imaging using the integrated transcapillary-resembling microfluidic system**

Using a two-photon confocal microscope (LSM750, Zeiss, Germany) equipped with an incubation chamber and an automated (motorized) stage positioning system, time-lapse confocal fluorescence live-cell imaging was carried out over 7 days for all cell lines (βiPLCs and FSβiPLCs embedded in alginate in the microchamber of the microfluidic device. The images were acquired from five different positions of the microchamber every 5 minutes in an automated fashion over 160 h. The acquired images were analyzed in ImageJ and the apparent occupancy area of dead cells (AODC) corresponding to DAPI signal and the apparent occupancy area of live cells (AOLC) corresponding to GFP (live βiPLCs) or mCherry (live FSβiPLCs) signals were evaluated for each frame. Then, an averaged apparent cell viability index (CVI) was calculated for each frame as:

$$CVI\% (t)=\frac{{\sum_{i=1}^{N=5} AOLC (i,t)}/N}{{\sum_{i=1}^{N=5} (AOLC \left( i,t \right)+AODC \left( i,t \right))}/N}\times100$$

where *i* is the image number and *N* is the total number of images acquired from 5 different positions of the microchamber at time *t*.

**Development of a perfusion GSIS assay on-a-chip**

We developed a PDMS-based microfluidic GSIS assay on-a-chip using soft photolithography and photolithography techniques (Supplementary Fig. 2a). The device was designed in AutoCAD, comprising a 1.5-m microchannel (800 μm wide, 80 μm high) and three switchable inlets (Supplementary Fig. 2c). The AutoCAD design was converted to photomasks using a laser pattern generator. followed by creating the SU8 master mold. The negative photoresist (SU-8 3000) was spin-coated onto a silicon wafer followed by baking for 10 min at 65°C and 45 min at 95°C. The SU8 was cured by UV exposure onto the photomask aligned with the baked photoresist (Supplementary Fig. 2a). After developing the SU8 master mold, we used the master mold and fabricated the microfluidic device by pouring PDMS on the mold and cure it for 2 h at 85°C. The PDMS microfluidic devices were cut and placed on plasma-treated glass slides followed by conditioning at 85°C overnight.

**Preparation of GSIS assay on-a-chip for Insulin secretion measurement**

The microfluidic device was coated by injecting Geltrex (ThermoFisher Scientific) into microchannels followed by washing with CellAdhere™ Dilution Buffer (STEMCELLTM Technology, Canada). Each cell type (βiPLCs, FSβliPLCs) was then gently introduced into the microchannels 3×10^6^ cells/mL using a 1 mL syringe. The device was incubated at 37°C for 20 minutes. Next, non-adherent cells were washed out by connecting the device to a syringe pump, injecting fresh culture medium into the microchannels at 15 μL/min for 15 minutes. Using a phase-contrast microscope, cell-seeded microchannels were imaged at eight different locations. Images were analyzed in ImageJ to determine the density of adhered cells per unit area of microchannels (Supplementary Fig. 2d).

**Computational Fluid Dynamics modeling of the GSIS assay on-a-chip**

Using COMSOL Multiphysics® v. 4.4 (COMSOL Inc., Burlington, MA) and applying initial and boundary conditions (Suppl. Fig. 6e), we solved fluid dynamics, mass transfer, and Insulin secretion kinetics equations for perfusion GSIS on-a-chip. Computations utilized the Pardiso direct linear solver with a maximum time-step of 0.5 s, ensuring reasonable computation times. Time-dependent boundary conditions at the inlet mimicked LG and HG solution flows. Predicted and experimental Insulin content values at the microdevice outlet were normalized based on cell density per microchannel area and total Insulin content over 100 min. A secretion rate index was defined as the percentage of Insulin secretion per minute relative to the total possible secretion over 100 minutes. The model was validated against measured values (Supplementary Fig. 4) and used to estimate the transition state during LG to HG inlet flow switch, optimizing sampling intervals based on predicted values.
