# Supplementary material for: Microfluidic chip systems for characterizing glucose-responsive insulin-secreting cells equipped with FailSafe kill-switch
Source: Stem Cell Res Ther. 2024 Dec 18;15:486. doi: 10.1186/s13287-024-04059-7 (PMC11656860; doi:10.1186/s13287-024-04059-7)
Supplement: Supplementary file 9 — Additional file 9. Supplementary Table: The list of primers used for RT-qPCR and genomic PCR. [file 13287_2024_4059_MOESM9_ESM.docx]

| **Supplementary Table 1.** The list of primers used for RT-qPCR and genomic PCR. | | | | | | |
| --- | --- | --- | --- | --- | --- | --- |
| Primer Name | Forward Sequence | | Reverse Sequence | PCR product expected size | | |
| *Insulin1* | GCCCTTAGTGACCAGCTATAATCAG | CTGTTGGTGCACTTCCTACCC | | | RT-PCR primers |  |
| *NKX6.1* | TCCTTGATCTGTGTCCTGCG | AGCCATTGGCACGATGGTTA | | | RT-PCR primers |  |
| *PDX1* | CCTCTGAAACCCGCAAGGAT | CGCAAGGAGGGAAGAGTGTT | | | RT-PCR primers |  |
| *HSV-TK* | CGAGACCATCGCCAACATCTA | GGCGTAGGGCATTCCCATT | | | RT-PCR primers |  |
| *Cdk1-TK* (5' junction) | TGATGGATTCGGGCAAGTTTCT | GTATCTGGCGGCAGGGTAGC | | | 1457 bp |  |
| *Cdk1-TK* (3' junction) | ATGGCCACAACCATGGTGAGCAAG | TGATGGATTCGGGCAAGTTTCT | | | 1616 bp |  |
| wild type *Cdk1* | TGATGGATTCGGGCAAGTTTCT | TGATGGATTCGGGCAAGTTTCT | | | 1711 bp |  |
